# Imaging beyond the surface region: Probing hidden materials via atomic force microscopy

**DOI:** 10.1126/sciadv.adg8292

**Published:** 2023-06-28

**Authors:** Amir Farokh Payam, Ali Passian

**Affiliations:** ^1^ Nanotechnology and Integrated Bioengineering Centre, School of Engineering, Ulster University, Belfast, UK.; ^2^ Quantum Computing and Sensing, Oak Ridge National Laboratory, Oak Ridge, TN 37830, USA.

## Abstract

Probing material properties at surfaces down to the single-particle scale of atoms and molecules has been achieved, but high-resolution subsurface imaging remains a nanometrology challenge due to electromagnetic and acoustic dispersion and diffraction. The atomically sharp probe used in scanning probe microscopy (SPM) has broken these limits at surfaces. Subsurface imaging is possible under certain physical, chemical, electrical, and thermal gradients present in the material. Of all the SPM techniques, atomic force microscopy has entertained unique opportunities for nondestructive and label-free measurements. Here, we explore the physics of the subsurface imaging problem and the emerging solutions that offer exceptional potential for visualization. We discuss materials science, electronics, biology, polymer and composite sciences, and emerging quantum sensing and quantum bio-imaging applications. The perspectives and prospects of subsurface techniques are presented to stimulate further work toward enabling noninvasive high spatial and spectral resolution investigation of materials including meta- and quantum materials.

## Teaser

The future of nanometrology lies beneath the surface, driving an exciting era of non-invasive, high-resolution investigation.

## INTRODUCTION

**Fig. 1.**
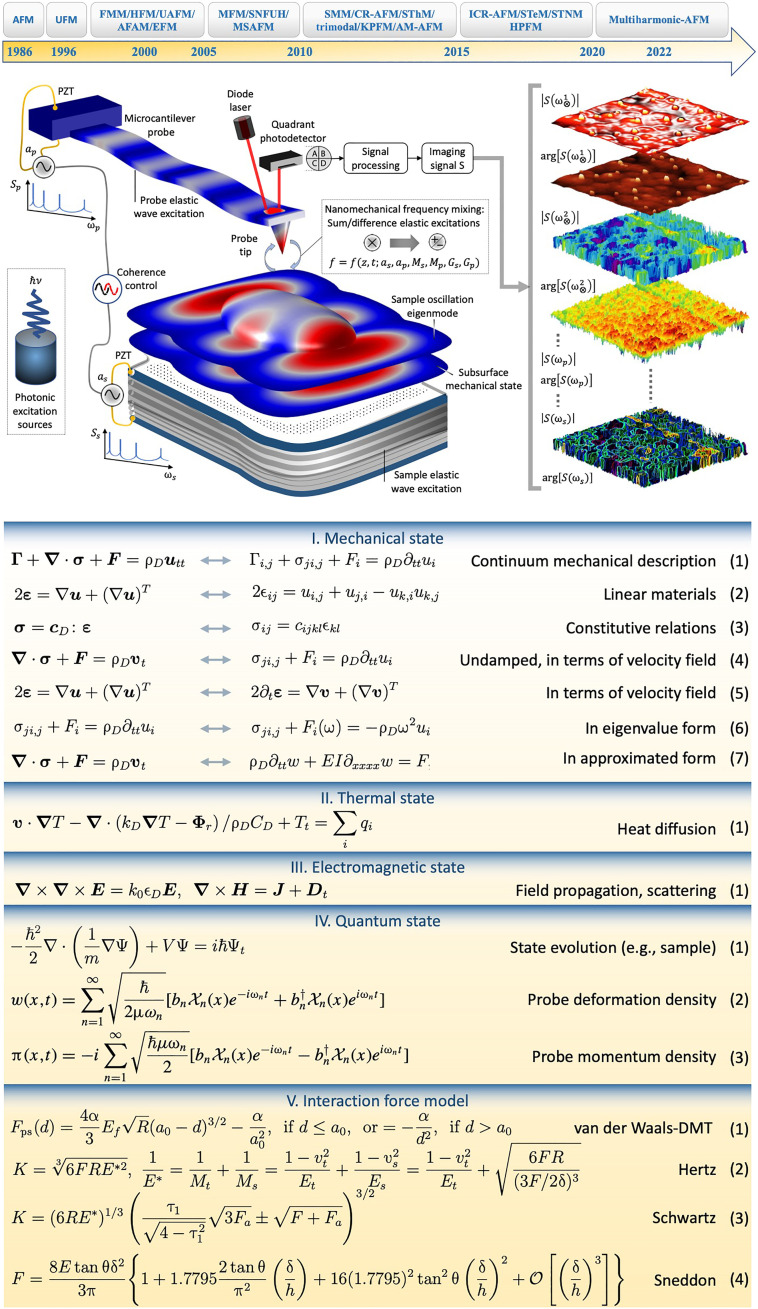
Subsurface physics. Top: Timeline of key inventions in subsurface force microscopy (FM): atomic FM (AFM), ultrasonic FM (UFM), force modulation microscopy (FMM), heterodyne FM (HFM), ultrasonic AFM (UAFM), atomic force acoustic microscopy (AFAM), electrostatic FM (EFM), magnetic FM (MFM), scanning near-field ultrasonic holography (SNFUH), mode-synthesizing AFM (MSAFM), scanning microwave microscopy (SMM), contact resonance AFM (CR-AFM), scanning thermal microscopy (SThM), kelvin probe FM (KPFM), amplitude modulation AFM (AM-AFM), intermittent contact resonance AFM (ICR-AFM), scanning thermoelectric microscopy (STeM), and scanning thermal noise microscopy (STNM). Middle: Schematic modalities where a probe (*p*), a sample (*s*), or both are excited by signals *S* of amplitudes *a* and adjustable frequency ω contents or coupled with specific properties of a specimen (e.g., photothermal or photoacoustic excitation by a photon of energy *h*υ). Bottom: Main equations describing the states (I to IV) of the probe and/or the sample, and their mechanical contact (V).

Measuring the subsurface domain presents an inverse and often nonlinear problem. Therefore, to obtain any information on the interior of a material nondestructively, one can only make an analytical or statistical inference of the features that caused the signal. However, the minimally invasive, label-free, nanoscale characterization of the inner structures, interfaces, and organization of two-dimensional (2D) materials (e.g., graphene), quantum materials (e.g., complex oxides), topological (e.g., bismuth selenide), Perovskite (e.g., methylammonium lead halide), bandgap materials (e.g., photonic crystals and multilayers), plasmonic [e.g., core-shell nanoparticles (NPs)], and metamaterials (e.g., cloaking devices) is essential. Optical, confocal, transmission electron, and near-field scanning optical microscopies (*1*–*5*) have been proposed and used in applications such as molecular biology (*6*, *7*), toxicity (*8*), polymer and composite science (*9*, *10*), multilayer high-integration chips, and multicomponent semiconductor device fabrication (*11*, *12*). However, classical diffraction at increasingly smaller length scales, limits the conventional imaging of buried structures with nondestructive excitations, such as low-intensity light and acoustic waves (*13*–*15*). Although electron microscopy can provide nanoscale resolution (*2*, *16*), irradiation damage to the sample, especially for biological and organic composite materials, can be a problem. Scanning probe microscopy (SPM) can be a remarkable candidate for tackling forward and inverse subsurface problems. The advanced mechatronics of SPM probes, and the ability to functionalize the probe tips, make the herein-discussed modalities highly relevant to the creation and control of interfacial photonic and semiconductor qubits, and quasiparticles such as polaritons and skyrmions. Here, our goal is to survey the measurement modalities of potential for studying hidden materials (Fig. 1).

## MOTIVATION

Quantitative microscopy of materials beyond their bounding surfaces (*17*) aims to explore two general features: (i) morphological characteristics, e.g., organization, distribution, order, and arrangement of subsurface interfaces and boundaries such as a cell’s nuclear membrane (*18*), and (ii) the chemical and physical properties of the enclosed materials such as the elasticity of chromosomes (*18*). Understanding these features and their functional properties in performances of pristine materials and in operando devices has enabled recent discoveries in, for example, strain-tuned electronics (*19*), photo-capacitive/photo-faradic bioelectronics (*20*), and additively manufactured polymers and composites (*21*). In semiconductor science and technology alone, controlling the shape and order of materials (*22*), detection of defects such as buried voids and delamination or leakage for failure analysis (*23*), and mapping the scattering patterns around the defects and impurities (*24*) are all use-cases for subsurface nanometrology. For example, the positioning of dopant atoms and roughness of material layers—a hidden morphology attribute—can affect interactions with photons, phonons, and electrons, ultimately determining the functionality of semiconductor devices such as the electrical behavior of transistors. In 3D organic integrated circuits, which incorporate interconnect among the layers, confirming reliable metal connections at different layers is demanded (*11*). To optimize the integrated circuits and quantum devices, subsurface investigation of the charge carriers and their energy levels at interfaces of organic semiconductors and electrodes (*22*) is needed. Other use cases include the study of scattering and optical topological transitions occurring in layered metal-dielectric structures and nanowire arrays fabricated by hyperbolic metamaterials (*25*). Similarly, to detect defects in superconductors (*24*), probing the Fermi sheets from differential conductance maps is needed.

To fabricate micro/nanoelectromechanical systems, imaging of the group of ions, clusters, lattice defects, and crystal grains is necessary to reduce the pattern size and predict and avoid the failure mechanisms including stiction, wear, fracture, and fatigue. To enhance the efficiency and stability of graphene-based devices, the characterization of buried interfaces is needed (*24*).

In biology, exploring the interior of a cell or tissue is of great importance to elucidate the fundamental mechanism of cell functions and diseases as well as for studying the effects of nanomaterials on biological systems (*26*, *27*). Identification of NPs embedded into cells and other biological materials has extensive applications in drug delivery (*28*), dental materials (*29*), contrast agents of magnetic resonance imaging (*30*), and cancer treatment (*31*).

The combination of polymeric matrix and nanomaterials creates nanocomposites that exhibit enhanced mechanical strength, thermal and electrical conductivities, stiffness, and toughness. The dispersion mechanism and the distribution of NPs within polymer components affect the morphology and interfacial properties of produced composites. Carbon nanotubes (CNTs) (*32*, *33*), graphene (*34*), nanocellulose (*35*), and clays (*36*) have all been used as fillers in composite technologies, which could lead to the dispersion of several phases inside the matrix, altering the nanocomposite properties (*37*). If the fillers, in an aggregated phase, failed to bond to the matrix or if a failure occurs in the reinforcing phase when bonding appropriately with the matrix is required, then the composite fails to achieve the optimal design properties. Typically, a layer of polymer covers the filler phases, making an evaluation, using conventional surface imaging techniques, difficult. For example, to improve the performance of CNT nanocomposites, the 3D material morphology of CNTs in a polymer matrix is needed to study the process-structure-properties relationships.

## SPM SUBSURFACE PHYSICS AND MEASUREMENT TECHNIQUES

In basic SPM, a probe at a distance *d* ≫ *a*_0_ is brought into the nanomechanical regime of a specimen where it interacts with the surface via attractive (*d* ≳ *a*_0_) and repulsive (*d* < *a*_0_) forces. Here, *a*_0_ denotes an appropriate interatomic distance (~Å, e.g., 1.54 Å for carbon-carbon bond length) (Fig. 1). In practice, this interaction picture is modified in the presence of other nanoscale phenomena, e.g., due to electrostatic, hydration, and ionic interference. Both the probe and the specimen may be excited and, from the collective input stimuli, the nonlinear probe-sample force (*F*_ps_) synthesizes additional modes that carry subsurface information. Depending on the operational mode, and the excitation-detection mechanisms, the techniques explored for subsurface visualization may be classified into six groups. These consist of mechanical, electrostatic, magnetic, electromagnetic, and thermal excitation of the probe, the sample, or both (Table 1), and the force-distance approaches (Fig. 1). The merits (including weaknesses and strengths), applications, and basic equations of these force microscopy (FM) modalities are presented in tables S1 and S2 and Fig. 1, respectively.

**Table 1.** Subsurface techniques and their spectral characteristics. AFM, atomic force microscopy; AFAM, atomic force acoustic microscopy; AM-AFM, amplitude modulation AFM; CR-AFM, contact resonance AFM; EFM, electrostatic FM; FMM, force modulation microscopy; HFM, heterodyne FM; HPFM, hybrid photonic-nanomechanical FM; ICR-AFM, intermittent contact resonance AFM; KPFM, kelvin probe force microscopy; LUFM, lateral ultrasonic FM; MFM, magnetic force microscopy; MSAFM, mode-synthesizing AFM; RDF-AFM, resonant difference-frequency AFM; SNFUH, scanning near-field ultrasonic holography; UAFM, ultrasonic AFM.

Technique	Sample drive frequency	Probe drive frequency	Detection frequency	Features
AM-AFM	–	*f*_1_	*f*_1_	Amplitude and phase versus indentation curves, the resonance frequency of the cantilever
AM-AFM dc-biased	–	*f*_1_	*f*_1_	The resonance frequency of the cantilever, phase lag, and tip dc bias
EFM (two steps)	–	*f*_1_	*f*_1_	The resonance frequency of the cantilever, electrostatic force, tip dc bias, and lift mode
EFM (one step)	–	*f*_1_ and *f*_elec_	*f*_1_ and *f*_elec_	The resonance frequency of the cantilever, electrostatic force, tip dc, and ac bias
KPFM	–	*f*_1_ and *f*_elec_	*f*_1_ and *f*_elec_	The resonance frequency of the cantilever, constant tip ac voltage, tip dc voltage for control purposes, and surface potential is the measured quantity
MFM	–	*f*_1_	*f*_1_	Resonance frequency of cantilever, magnetic force, phase, and/or frequency of cantilever in lift mode are used to construct magnetic images
Bimodal (trimodal) AFM	–	*f*_1_ (*f*_*c*1_), *f*_2_ (*f*_*c*2_),…	*f*_1_ (*f*_*c*1_), *f*_2_ (*f*_*c*2_),…	Resonance frequencies of the cantilever, simultaneous excitation and detection of different eigenmodes of cantilever, amplitude, and phase images
FMM	*f*_1_(*f_c_*)	–	*f*_1_(*f_c_*)	Low frequency, contact resonance, and linear repulsive regime of interaction
AFAM (CR-AFM)	*f*_1_(*f_c_*)	–	*f*_1_(*f_c_*)	High frequency, contact resonance, contact stiffness measurement, and acoustic frequency
UAFM	*f*_1_(≈*f_c_*)*	*f*_1_(≈*f_c_*)*	*f*_1_(≈*f_c_*)	Contact resonance, linear and/or nonlinear interaction regime, and ultrasonic frequency
Second-harmonic UAFM	*f*_1_(≈1/2*f_c_*)	–	2*f*_1_(≈*f_c_*)	Nonlinear force, contact resonance, and ultrasonic frequency
CR-AFM	*f*_1_ (*f_c_*)	–	*f*_1_ (*f_c_*)	Contact resonance, the sample is excited to excite and detect the *n*th contact eigenmode frequency of cantilever, simultaneous topography, and contact stiffness imaging
UFM	*f*_1_(≫*f_c_*), *f_m_* ≪ *f*_1_	–	*f_m_*	Nonlinear force, high frequency, nonlinear interaction regime through modulation frequency (*f_m_*), and ultrasonic response
LUFM	*f*_1_(≫*f_c_*),	*f*_2(torsional)_(≪*f_tc_*)	*f*_1_(≫*f_c_*), *f_tc_*	Nonlinear force, high vertical frequency, and torsional vibration
ICR-AFM	*f*_PFT_	*f*_1_ (*f_c_*)	*f*_1_ (*f_c_*), *f*_PTF_	Contact resonance, intermittent contact, measurement of contact stiffness at different indentation depths, the 3D elastic response of the material, characterization of adhesive property, and dynamics of the dissipated energy
HFM (on-resonance) (RDF-AFM)	*f*_1_ (≫*f_c_*)	*f*_2_ (≈*f*_1_ + *f_c_*)	∣*f*_2_ − *f*_1_∣ (≈*f_c_*)	Nonlinear force, contact resonance, high frequency, amplitude, and phase at the beat frequency
HFM (off-resonance)	*f*_1_ (≫*f_c_*)	*f*_2_ (≈*f*_1_ + *f_c_*)	∣*f*_2_ − *f*_1_∣ (≠ *f_c_*)	Nonlinear force, high frequency, amplitude, and phase at the beat frequency
SNFUH	*f*_1_ (≫*f_c_*)	*f*_2_ (≫*f_c_*)	∣*f*_2_ − *f*_1_∣	Soft-contact mode (for hard specimen) and near-contact mode (for soft samples), holography and near-field mode for detection, and noninvasive acoustic waves for depth sensitivity
MSAFM	*f_i_* (*i* = 1:*n*)	*f_j_* (*j* = 1:*m*)	∣*f_i_* − *f_j_*∣	Coupled mode at various frequencies corresponding to a synthesized mode
			*i* = 1:*n*, *j* = 1:*m*	
HPFM	*f_i_* (*i* = 1:*n*), *hν*	*f_j_* (*j* = 1:*m*)	∣*f_i_* − *f_j_*∣	Hybrid (nanomechanical + photonic) approach using a synthesized mode in conjunction with infrared (*hν*) photoacoustic spectroscopy (chemical mapping)
			*i* = 1:*n*, *j* = 1:*m*	

*In UAFM, the cantilever (*46*) or sample (*62*) can be excited.

In the equation panel of Fig. 1, vectors ***u*** = (*u*, *v*, *w*) and **σ** denote the displacement and stress fields, respectively, within a sample or a probe (with density ρ) that is subjected to a volume force ***F*** and a general damping **Γ**. The solution of the (Navier) equation of motion (Eq. I.1) yields the system’s mechanical state. The strain field **ε** is proportional to **σ** via the material properties of domain *D* given by tensor *c_D_* (e.g., with components proportional to Young’s modulus *E*). When possible, Eq. I.1 could be simplified for a probe with a beam shape (having a second moment of area *I*). The temperature of a domain with heat capacity *C_D_*, thermal conductivity tensor *k_D_*, and a tensor dielectric function ϵ*_D_* could be altered, for example, via inelastic scattering of the photons of wave vector *k*_0_ in applied fields (***E*,*H***). Losses such as nonradiative decay due to photo-absorption and/or thermoelastic effect due to the piezoelectric drive (PZT) furnish Eq. II.1 with source terms *q_i_*. For a radiative heat flux Φ*_r_* and a domain translational motion velocity vector ***v***, the thermal state *T* is the solution of Eq. II.1. Light scattering resulting in an electric displacement field ***D*** together with current ***J*** leaves the system in an electromagnetic state described by the solution of Eq. III.1. The quantum state (e.g., charge states) ψ of a nanoscale specimen such as shallow and bulk defects in crystalline materials (e.g., nitrogen-vacancy centers in diamond) or free electrons confined in a matrix (e.g., quantum dots in a semiconductor) could be obtained from Eq. IV.1 if the potential *V* and the effective mass *m* are known. The probe could also be prepared in a mechanical quantum state (e.g., squeezed deformation state of a silicon microcantilever or a shallow ion entangled with a mechanical eigenmode of the cantilever). The displacement *w* and momentum π densities of the cantilever, as sums over eigenstates of frequencies ω*_n_*, are given by Eqs. IV.2 and IV.3, respectively. A challenge in quantitative imaging is the lack of unified and accurate tip-sample contact mechanics (*38*). The main common models displayed in Eqs. V.1 to V.4 are used to quantify material properties, though reproducibility issues remain. The properties enter these models via effective tip-sample stiffness *E_f_*, Hamaker constant *H* (via α = *HR*/6), and Poisson ratio υ. For a tip of a given shape (e.g., conical, pyramidal, and parabolic), a radius of curvature *R* (~10 nm) and half-angle opening θ are used together with an indentation depth δ occurring at a sample point of height *h*, in response to a force *F* to yield a stiffness *K*.

## PROBE MECHANICAL EXCITATION

**Fig. 2.**
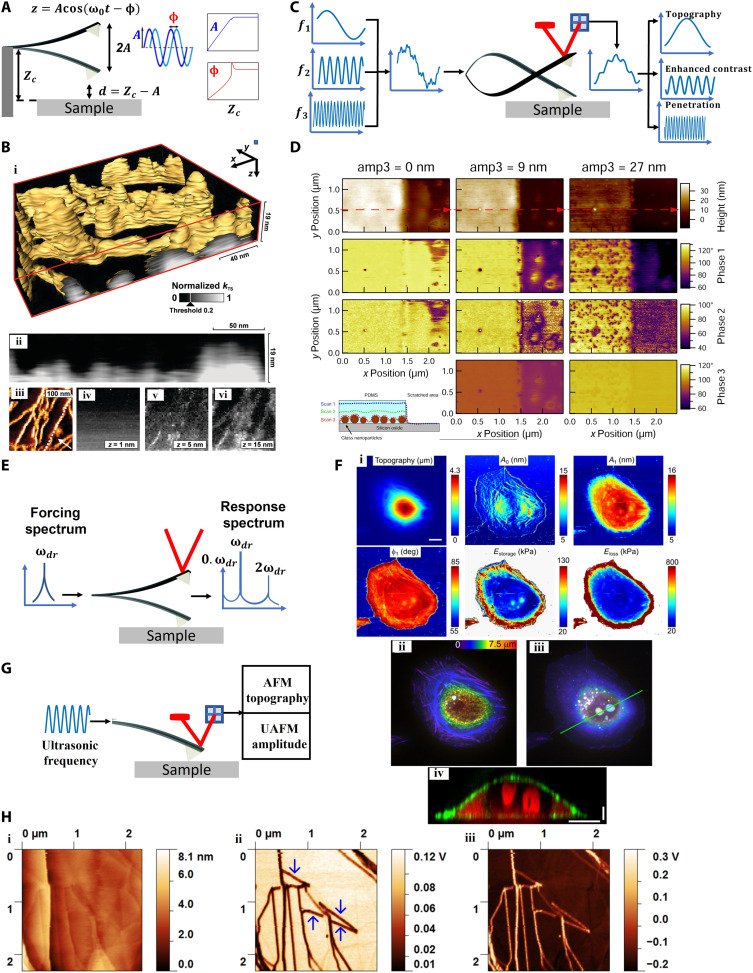
SPM-based probe mechanical excitation for subsurface imaging. (**A**) Principle of tapping-mode (amplitude modulation) AFM. (**B**) (i) Three-dimensional reconstructed stiffness of the top 19 nm of erythropoietic protoporphyrin with a single slice shown in (ii), and along a lamella marked with the dotted line in the phase image (acquired at 90% amplitude setpoint) (iii), where the crystalline lamellae and amorphous material are represented by bright and dark regions, respectively. (iv to vi) Representative stiffness maps at different indentation depths of 1, 5, and 15 nm, respectively [adapted with permission from Spitzner *et al*. (*9*)]. (**C**) Principle of bi/trimodal AFM. (**D**) Topography and phase-shift imaging invoking the first, second, and third eigenmode reveal buried glass NPs under spin-coated polydimethylsiloxane (PDMS) film on SiO_2_ substrate, as shown for various third mode amplitude setpoints [adapted with permission from Ebeling *et al*. (*40*)]. (**E**) Driving the cantilever at ω*_dr_*, creates oscillation harmonics *n*ω*_dr_* (*n* = 0,2,3, ⋯) due to the tip-sample force nonlinearity. (**F**) (i) A single breast cancer cell (MDA-MB-231), and color-coded height maps of scanning droplet cell microscopy (ii and iii) where the amplitude, phase, and local storage of Young’s modulus reveal peripheral actin stress fibers and nucleoli. The topography and deflection maps could only visualize the actin fibers. (iv) A slice along the green line in (iii) shows the depth and size of the nucleoli (red) and cell shape and F-actin (green) [adapted with permission from Efremov *et al*. (*45*)]. Scale bars, 10 μm in the horizontal direction and 2 μm in the vertical direction. (**G**) Probe excitation in UAFM. (**H**) UAFM-generated surface/subsurface images of graphite. Topography (i), amplitude (ii), and phase (iii) maps. Buried features (arrows) could only be observed in amplitude and phase images [adapted with permission from Wang *et al*. (*46*)].

The most straightforward atomic FM (AFM) modality that could provide a subsurface channel involves mechanical excitation of just the probe at specific frequencies (see “Mechanical state” in Fig. 1). The cantilever response (photodetector output signal) is detected at the same frequencies. Amplitude modulation AFM (AM-AFM), also called tapping mode, is the most widely used AFM technique in which a cantilever is oscillated at or near its natural resonance frequency while the oscillation amplitude is kept constant by adjusting the tip-sample height via a feedback controller (*39*). The topographic information is acquired from the feedback loop, while the phase shift between the cantilever excitation and the response detection provides information about the mechanical properties of the specimen (Fig. 2A). The tip indentations and amplitude/phase versus distance curves, captured during the approach to and retract from the specimen, may be used to reconstruct 3D images, for example, of the block copolymer and semicrystalline samples, as shown by Spitzner *et al.* (*9*) (Fig. 2B). By determining the indentation into the specimen and using the effective spring constant, and the dissipation and damping quantities that describe the tip-sample interaction, a depth-resolved image may be obtained (*9*). Because of the necessity of collecting amplitude/phase data at different points of the sample for different indentation depths, the data acquisition and analysis are time-consuming and complex in this method (see table S1).

Subsurface characterization may also be obtained from bimodal/trimodal AFM (*40*, *41*), in which the cantilever is simultaneously vibrated by two or three driving forces at first and second (and third) eigenfrequencies (see the eigenvalue form of the “Mechanical state” in Fig. 1) of the cantilever (*42*). The amplitude or the frequency shift, depending on the first mode modulation scheme [amplitude modulation or frequency modulation], is used for topography, while the second (and third) modes are used to measure the mechanical, electrical, or magnetic properties (Fig. 2C) (*43*, *44*). Using trimodal AFM, Ebeling *et al*. (*40*) simultaneously imaged the topography and the subsurface compositional contrast and quantified the depth of buried NPs inside a soft matter (Fig. 2D). In this trimodal approach, the first eigenmode of the cantilever is used for topography, while the compositional contrast and indentation depth are acquired from the second and third eigenmodes, respectively. The results demonstrated that increasing the setpoint amplitude of the third mode leads to the detection of more internal features from the phase shift of the first and second eigenmodes. A 30-nm lateral resolution was reported in this study. Perrino *et al*. (*41*) applied trimodal AFM with a sub-10-nm lateral resolution to image iron oxide NPs and silicon nanowire (Si-NW) circuits embedded under a 70-nm layer of polydimethylsiloxane. Both topography and phase contrast of the first mode could detect the spin-coated Si-NWs and iron oxide NPs.

Recently, Efremov *et al*. (*45*), using multi-harmonic AFM with a long-tip microcantilever, imaged the 3D subcellular and subnuclear structures of living cells (Fig. 2, E and F). In multi-harmonic AFM, the cantilever is directly excited by magnetic, Lorenz, piezoelectric, or photothermal forces. Observables, including the amplitudes and phases of zeroth, first, and second harmonics of the cantilever, are then acquired and linked to appropriate contact mechanics models (e.g., Hertz or Sneddon) to associate the observable parameters with the surface and subsurface properties of specimens (*45*).

In ultrasonic AFM (UAFM) (*46*, *47*), the cantilever or sample oscillation, caused by external forces, is detected at the contact resonance (CR) frequency (*f_c_*), while the interaction may be controlled to be in both linear and nonlinear regimes (Fig. 2G). To maintain the system in a linear regime, the frequency of detection equals that of actuation. However, if the UAFM detection is performed by a demodulation scheme, then the force applied on the tip is considerably higher and the system is in the nonlinear regime. UAFM has been used to detect defects in graphite within the range from 18.6 to 77.1 nm (Fig. 2H) (*46*).

## SAMPLE MECHANICAL EXCITATION

**Fig. 3.**
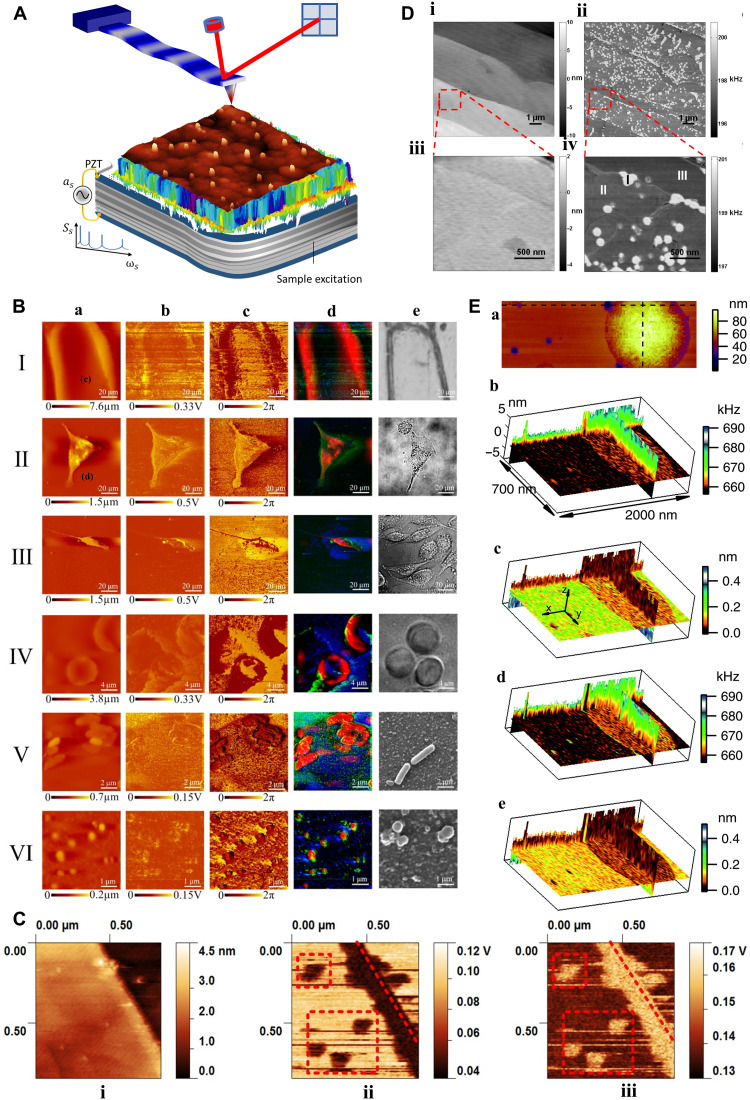
SPM-based sample mechanical excitation for subsurface imaging. (**A**) The specimen is driven at acoustic or ultrasonic frequencies, while the probe remains engaged with the surface. (**B**) Columns a to e show morphology, amplitude, phase, fused, and reference maps of eukaryotes and prokaryotes cells. Row I: Onion epidermal cells; II: MCF7; III: MDA-MB-231; IV: human erythrocyte; V: *Escherichia coli*; and VI: *Staphylococcus aureus* cells [adapted with permission from Li *et al*. (*54*)]. (**C**) Defect detection in graphite using AFAM topography (i), amplitude (ii), and phase (iii). The demarcated features in the amplitude and phase maps are absent in the topography. The dashed lines indicate the edge position in the height map [adapted with permission from Wang *et al.* (*46*)]. (**D**) CR-AFM image on an oxygen-intercalated multilayer graphene sample discloses recognizable subsurface regions. (i) Topography and (ii) associated CR frequency imaging of the first flexural mode. (iii and iv) Zoom-in height and frequency maps of the regions illustrated in the red box in (i) and (ii). CR-AFM discloses three recognizable areas highlighted by “I,” “II,” and “III” in (iv) [adapted with permission from Tu *et al*. (*65*)]. (**E**) Cross-sectional tomography with CR-AFM. (a) AFM micrograph illustrating the surface height of a polystyrene-polypropylene (PS-PP)–blend polymer. (b and d) Cross-sectional tomography maps of CR frequency and (c and e) the second eigenmode amplitude across the region in (a). The vertical *xz* and *yz* cross sections of the tomography maps alongside the crosslines are illustrated in (a). The maps in (b) and (c) were extracted from captured data during probe approaches toward the specimen, and the maps in (d) and (e) were extracted from captured data during retracts from the specimen. The PP and PS areas have been determined visually in the horizontal tomography planes by their contrast in frequency and amplitude [adapted with permission from Stan *et al*. (*66*)].

Subsurface information may also be garnered, by mechanical excitation of the sample, typically, at frequencies in the kilohertz, acoustic, and ultrasonic ranges (Figs. 1 and 3A). The elasticity and dissipation energy at the tip-sample contact point carry subsurface information. This is used in force modulation microscopy (FMM) (*48*, *49*), where the tip maintains contact with the specimen in the repulsive regime of the interaction while being driven through sample vibration at a few kilohertz. FMM has been used for subsurface imaging of the core of polymer-encapsulated cobalt NPs (*49*). However, to avoid any tip or sample damage, the materials used in FMM measurements are to have stiffness values in the order of cantilever spring constant, which is generally low (see table S1).

FMM-like techniques may be used to also detect MHz sample vibrations. As in FMM, the tip-sample distance modulation in the repulsive (linear) regime of the interaction causes a cantilever oscillation at the sample frequency. These high-frequency techniques are used in atomic force acoustic microscopy (AFAM) (*50*) and scanning local acceleration microscopy (*51*), which, compared to FMM, offer the advantage of using compliant cantilevers to attain contact stiffness of stiff materials. The remarkable reduction in friction caused by the high-frequency vibration decreases the risk of damage when imaging soft delicate samples. AFAM has visualized gold NPs buried in polystyrene (PS) films, gold lines covered by polymethyl methacrylate (PMMA) (*52*), defects in amorphous films (*53*), subcellular features (Fig. 3B) (*54*, *55*), and defects in graphite (Fig. 3C) (*46*).

In ultrasonic FM (UFM), a sample is excited at ultrasonic frequencies, which are higher than the resonance frequencies of typical cantilevers. As the cantilever cannot respond resonantly to the sample vibration, detection in the linear regime is less useful. Therefore, if the tip-sample distance is modulated within the nonlinear regime (see “Interaction force model” in Fig. 1), then an additional ultrasonic force (averaged over a period) acts upon the cantilever (*47*, *56*–*58*), causing an additional displacement. Its magnitude is a function of the tip-sample interaction regime that is swept by the tip-sample distance while being modulated at ultrasonic frequencies. Hence, the cantilever deflection will depend on the sample surface and subsurface elastic and adhesive properties. Therefore, the subsurface features of different elasticity which are located within the range of the contact stiffness field can be imaged. Detection of the molybdenum disulfide (MoS_2_) and thin flakes of graphite that were transferred onto structured polymeric substrates (*56*), characterization of early and late-stage amyloid-β peptide aggregation (*47*), and observation of the lattice defects under atomically flat terraces of highly oriented pyrolytic graphite (HOPG) (*59*) are examples of UFM uses. van Es *et al*. (*60*) used the UFM to image aluminum nanofeatures embedded under 300-nm-thick photoresist, photoresist with titanium, and SiO_2_ layers, respectively. Recently, Piras *et al*. (*61*) have used UFM to image aluminum features on a silicon substrate embedded beneath a 300-nm-thick photoresist and 50-nm-thick titanium layers. In second-harmonic UAFM (*62*), a sample oscillates at a cantilever frequency, *f*_1_ (close to half-CR frequency, *f_c_*/2). Higher-order cantilever oscillation, excited by nonlinear interaction, can be monitored with high sensitivity at 2*f*_1_, which is close to *f_c_*. Using this mode of UAFM, Au particles embedded in the polymer top-coat film have been detected (*62*).

To measure its *n*th contact eigenfrequency, the cantilever is excited over a spectrum encompassing the *n*th and (*n + 1*)th of its free resonance frequencies through sample excitation (see “Mechanical state” in Fig. 1). With a known *n*th free resonance frequency, the CR frequency $f_{c}^{n}$ may be quantitatively associated with the contact stiffness of the tip-sample interaction, allowing topographic and contact stiffness imaging (*63*). Using CR-AFM, silica NPs buried in PS have been imaged at depths ranging from 32 to 165 nm (*63*). Topography and stiffness mapping of microglia cells engulfed with Fe_3_O_4_ NPs (*64*), detection of silica NPs embedded beneath PS films (*63*), and detection of defects, subsurface features, and atomic structures of graphene featuring deliberately modified subsurface interfaces are examples of CR-AFM (Fig. 3D) (*65*). Quantifiable CR-AFM has been used to extract details of surface and subsurface features of PS-polypropylene blends, in terms of dissipation and Young’s modulus (Fig. 3E) (*66*). Recently, CR-AFM has been used to discern the subsurface of flexible circuit samples with 52-, 117-, 185-, 380-, and 653-nm-thick top layers, respectively (*67*).

## INTEGRATED SAMPLE AND PROBE EXCITATION

**Fig. 4.**
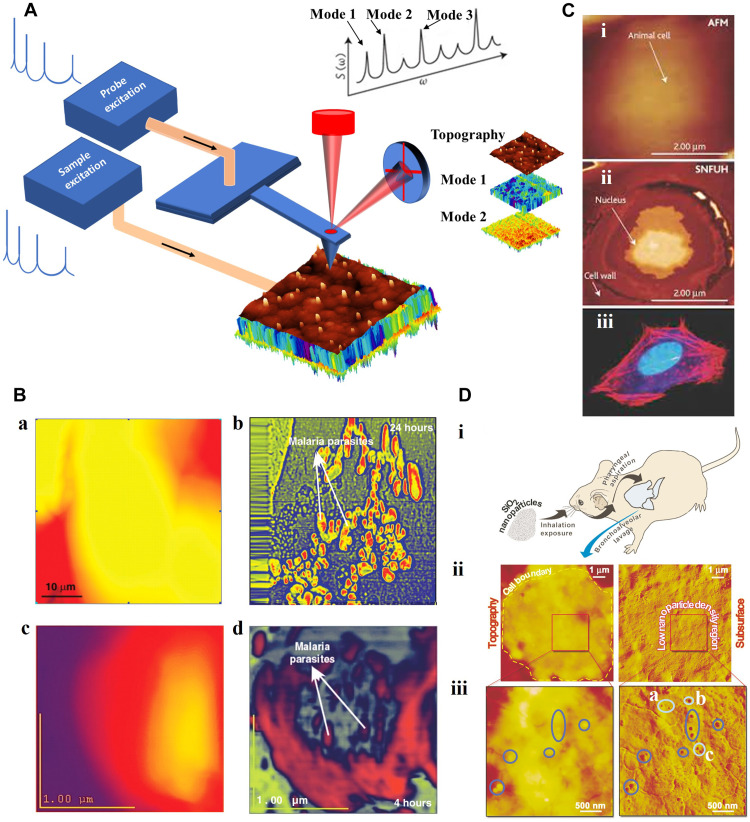
SPM-based integrated probe/sample mechanical excitation for subsurface imaging. (**A**) Schematic implementation of full-frequency excitation. (**B**) AFM topography (a and c) and SNFUH phase maps (b and d) reveal (after 24-hour incubation) features of malaria-infected red blood cells and a notable contrast due to parasites inside. (c) and (d) illustrate early-stage parasite infection after 4 hours of incubation [adapted with permission from Shekhawat and Dravid (*73*)]. (**C**) (i) Mouse cell topography and (ii) intracellular imaging using SNFUH. (iii) Micrograph of mouse fibroblast cell (blue: nucleus, red: actin protein in cell’s skeleton) [adapted with permission from Diebold (*74*)]. (**D**) Topography and detection of intracellular NPs embedded in alveolar macrophages from SiO_2_-exposed mice. The exposure route and cell sample origin are shown in (i). Topographic and SNFUH phase [(ii) and (iii) (left and right, respectively)] maps from a cell collected 24 hours after exposure. (iii) A chosen area in (ii) is rescanned at a higher resolution, where ovals indicate the existence of silica. The dark blue ovals show SiO_2_ that resides on or under the cell membrane, while a, b, and c illustrate NPs deep inside the cell (*27*).

A more diversified subsurface signal transduction may be obtained via excitation of both the cantilever and the sample at frequencies encompassing the cantilever resonance spectrum (~ kHz) up to ultrasonic ranges (Fig. 4A). Under such an excitation scheme (see “Interaction force model” in Fig. 1), for example, the cantilever torsional vibration can provide the map of subsurface features including delamination or edge dislocations (*58*). To achieve this, the sample can be driven laterally at frequencies below the cantilever resonance frequency. As a result, the cantilever can be excited into a torsional mode via surface friction forces, a technique referred to as lateral force modulation. Excitation of additional vertical ultrasonic oscillation of the sample changes the torsional torque of the cantilever during the tilt of the tip (*58*). As the torsional torque is generated during tip indentation into the sample, it is sensitive to surface friction and subsurface shear rigidity.

The simultaneous mechanical stimulation of the probe and the sample furnishes a dynamic platform for subsurface microscopy (*68*). In heterodyne FM (HFM), the sample and the cantilever are excited at different ultrasonic frequencies, whereas, in resonant difference-frequency atomic force ultrasonic microscopy (RDF-AFUM), the difference is tuned to the resonance frequency of the cantilever (*69*–*71*). The nonlinear tip-sample interaction leads to different frequency generation. As the specimen is driven at the frequency *f*_1_ and the cantilever at *f*_2_, the cantilever oscillation is modulated at *f*_1_ − *f*_2_ (beat frequency). If the total amplitude is sufficiently high for covering a nonlinear range of interaction force, then an ultrasonic force acts upon the cantilever and causes cantilever oscillation at different mixed frequencies. In HFM, this oscillation can be lock-in detected in amplitude and phase, exploiting the electronically mixed signal as a reference. A distinctive characteristic of HFM is its capability to detect the nonlinear phase shifts between tip and sample with high temporal sensitivity. Minute differences in the sample viscoelastic/adhesive response to the tip interaction result in a phase shift of the beat signal that could be probed in the phase of HFM. The information furnished by the amplitude of HFM is very similar to UFM. Whereas, in HFM, both the cantilever and sample oscillate, in UFM, the modulation frequency is selected below the CR frequency. Using HFM, the stiffness and viscoelastic properties of PMMA rubber nanocomposites were measured (*72*). Also, Kimura *et al*. (*62*) used HFM to image Au NPs buried in a photopolymer.

To image subsurface details of both soft and hard materials, a modality similar to HFM, RDF-AFM, and UFM (*47*, *69*, *71*), the scanning near-field ultrasonic holography (SNFUH) was developed (see table S1 for comparison) (*73*, *74*). SNFUH has been used to image NPs in cells (*26*, *27*). In SNFUH, the sample and cantilever are driven at megahertz frequencies, that is, notably higher than those of the cantilever resonances. Like x-ray standing waves which form by the interference of scattered and reference x-ray waves, a surface acoustic standing wave is formed from the interference of the two waves in SNFUH. In this picture, a lock-in amplifier monitors the perturbations in the phase and amplitude of the surface acoustic standing waves. Hence, when the sample acoustic response is perturbed by internal features, the cantilever can monitor the resultant alteration in the phase. Imaging with SNFUH has been reported for gold NPs embedded under polyvinylpyrrolidone and malaria parasites within the red blood cells (Fig. 4B) (*73*), mouse cells (Fig. 4C) (*74*), and SiO_2_ NPs confined within a macrophage (Fig. 4D) (*27*).

**Fig. 5.**
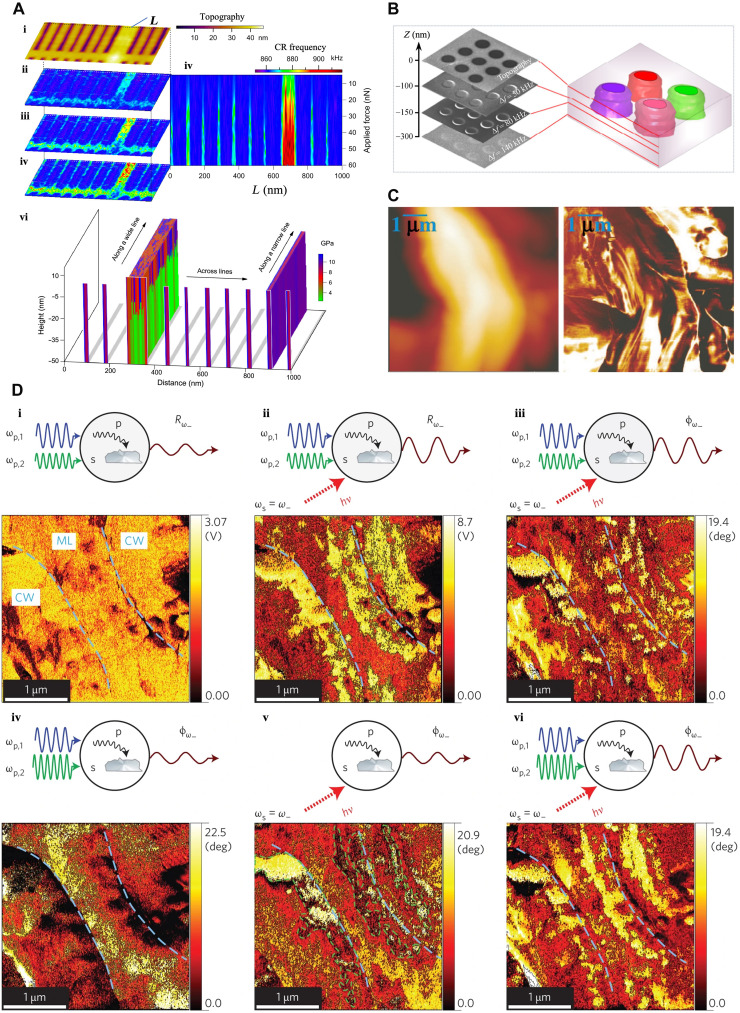
SPM-based integrated probe/sample mechanical excitation for subsurface imaging. (**A**) Topography (i) of patterns of narrow and wide fins, and the corresponding ICR-AFM constant-load tomographic sections (ii) to (iv) contact stiffness versus applied force. (v) CR frequency versus force along *L*. (vi) Calculated elastic modulus. Gray shows fin locations (identified from CR-AFM and AFM measurements on top of the fins) [adapted with permission from Stan *et al.* (*75*)]. (**B**) MSAFM phase images of PMMA calibration samples [adapted with permission from Vitry *et al.* (*14*)]. (**C**) MSAFM of poplar plants. Topography of the cell walls (left) and ultrastructure detected at a mixed frequency: sample driven at a single frequency and probe driven at two different frequencies (right) (*77*). (**D**) HPFM of biomass where *h*υ denotes the infrared photoacoustic excitation of the plant material (s), ω_−_ a difference frequency of the probe, and ω_s_ the specimen frequency. Probe amplitude (*R*_ω_) map without (i) and with (ii) *h*υ. Phase (ϕ_ω_) map with (iii) and without (iv) *h*υ. (v) Phase map with *h*υ but without mechanical actuation. The regions of decreased compositional contrast are depicted with green, dashed lines. (vi) Phase map with *h*υ and a higher probe drive amplitude. ω_−_ maps in the absence of *h*υ (i and iv) illustrate the sample morphology. Monochromatic maps (exploiting virtual resonance) provide chemical information (ii, iii, and vi). Tuning *h*υ discloses nanoscale areas rich in cellulose (iii and v) not probed without *h*υ (i and iv) or without mechanical actuation (v) (*80*).

We now consider an extension of the CR-AFM, specifically, the intermittent CR AFM (ICR-AFM) (*15*, *27*). Here, peak force tapping (PFT), i.e., force-frequency measurements throughout individual oscillations of an intermittent-contact AFM mode, may be used to map the subsurface variations of the nanomechanical properties of high aspect ratio low-k-dielectric patterns (small relative dielectric constant) across 20- and 90-nm-wide patterns (Fig. 5A) (*75*). In ICR-AFM, as the tip is tapping the sample, the induced variation in the resonance frequency of a cantilever eigenmode is measured progressively. As PFT is a force-controlled AFM mode, there is a possibility of synchronization of frequency measurements with the applied force during tip-sample interaction, which can lead to a more accurate value of the contact stiffness. ICR-AFM enables (i) measurements of contact stiffness at different indentation depths that yield 3D elastic response imaging, (ii) probing the details of the tip-sample interaction at contact formation and breaking that yield characterization of the sample adhesive property, and (iii) measurements of dissipated energy during tip-sample interaction, either in contact or out of contact. Using ICR-AFM, the adhesion, elasticity, and dissipation maps of PS/PMMA film contains submicrometer-size PMMA domains were captured (*76*).

Given the many AFM multifrequency modalities, the question of whether it would be possible to unify the described methods is prudent. The mode-synthesizing AFM (MSAFM) (*77*) was developed to answer this question. In MSAFM, both the cantilever and sample are allowed to be driven with arbitrary waveforms. In general, the nonlinear nanomechanical coupling of the cantilever and the sample causes remarkable frequency mixing. The excitations of probe and sample allow a broad spectrum of first- and higher-order couplings to be explored, providing a multitude of additional information channels for FM. The amplitude or phase map of the cantilever motion, detected at specific mixed frequencies, reveals the heterogeneity in the volume of the material underneath the AFM tip (*77*). MSAFM has imaged SiO_2_ NPs in cells (*78*), gold NPs in Ni (*79*), carbon nanohorns in alveolar macrographs and red blood cells (*78*), structure and composition of PMMA/exposed PMMA dielectrics at different depths (Fig. 5B) (*14*), and (poplar) plant cell walls ultrastructures (Fig. 5C) (*27*, *77*, *78*). In MSAFM, when an excitation channel is furnished photonically rather than mechanically, hybrid photonic-nanomechanical FM (HPFM) is achieved, allowing for spectroscopic interrogation. The HPFM (*80*) offers two features: (i) the generation of sum and difference frequencies due to the nonlinear nature of tip-sample interaction as a function of tip-sample distance and (ii) the dynamic influence of the so-called virtual resonance. In the latter, the oscillation at any generated mixed frequency can be magnified. In HPFM, the forces needed to drive the probe, the sample, or both can be garnered either elastically through a piezoelectric transducer or photoacoustically by the accessible photon absorption bands of the sample (typically via infrared photothermal absorption). Thus, to simultaneously obtain high spatial and spectral resolutions, HPFM uses a photonically induced sample vibration that takes part in frequency mixing. This method can be used in cancer research, nanotoxicity, and energy storage/production (Fig. 5D) (*80*).

## MAGNETIC, ELECTROSTATIC, AND ELECTROMAGNETIC EXCITATION

**Fig. 6.**
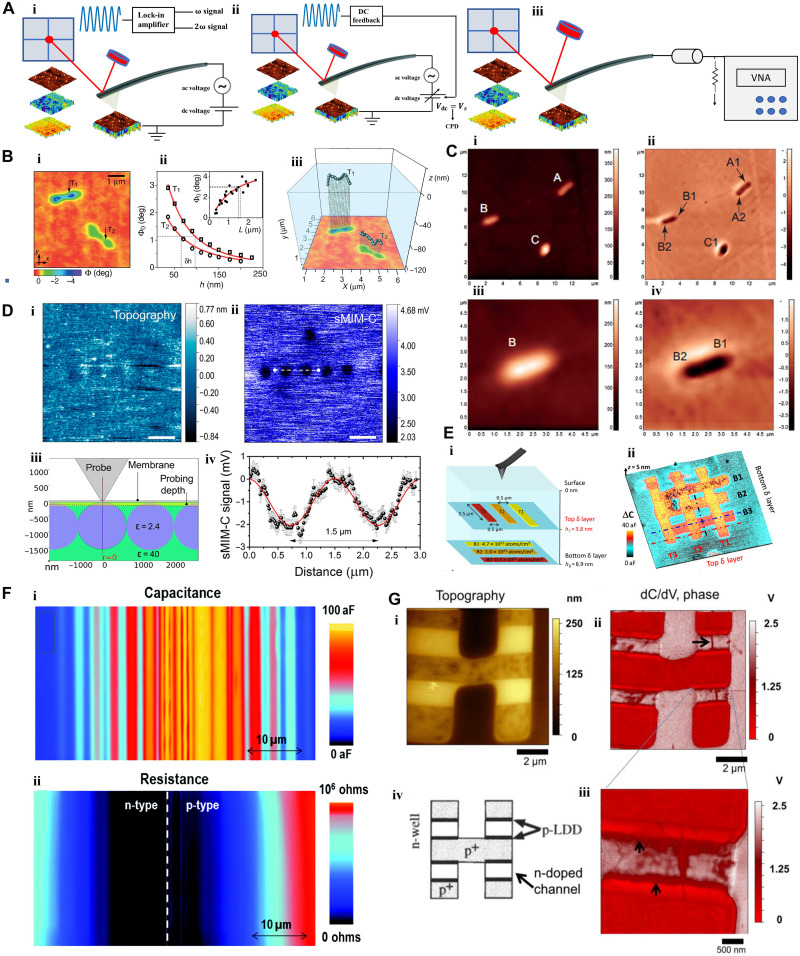
SPM-based electromagnetic excitation for subsurface imaging. (**A**) General schematic of (i) EFM, (ii) KPFM, and, (iii) scanning microwave microscopy. (**B**) EFM of 170-nm-thick film of single-walled CNT (SWCNT)/PMMA composite revealing two hidden SWCNTs (i), showing (points T_1_ and T_2_) lift height dependence on length-corrected signal (ii), and the 3D reconstructed image of the two nanotubes (iii) (blue: PMMA matrix) [adapted with permission from Jespersen and Nygard (*83*)]. (**C**) MFM, visualizing three nonspherical aggregates (A, B, and C) of noisome (i) with remarkable phase contrast between substrate and vesicles. Topography and phase images show vesicle B resulted from the coalescence of two vesicles B1 and B2 containing many magnetic NPs [adapted with permission from Dong *et al*. (*92*)]. (**D**) Topography (i) of a 50-nm-thick SiN membrane, below which PS particles in glycerol are revealed (ii) by capacitive-mode scanning microwave impedance microscopy (sMIM) [probing depth identified in (iii)] at a scale bar of 4 μm [adapted with permission from Tselev *et al*. (*99*)]. For clarity, the plot in (iv) displays the signal along the white dashed line in (ii). (**E**) (i) Sketch of a 3D Si structure encompassing three phosphorous bars with increased dosing (coded by the same colors for both layers) at two different heights. SMM capacitance map (ii) shows the δ-layer structure with ∆ωC and ∆G line profiles [adapted with permission from Gramse *et al*. (*93*)]. (**F**) SMM capacitance image of bipolar-doped silicon sample [adapted with permission from Brinciotti *et a*l. (*101*)]. (**G**) SMM topography (i) and capacitance gradient (ii and iii) images of the p-n junction (iv) [adapted with permission from Huber *et al.* (*103*)].

In addition to the explicitly mechanical actuation of the probe-sample system (including the photoacoustic actuation in HPFM), other material responses such as electrostatic, magnetic, or electromagnetic, stimulated through sending voltage or microwave signals to the cantilever and/or sample, are of interest in our review of subsurface approaches (see “Electromagnetic state” in Fig. 1 and table S1). Using AM-AFM in conjunction with an applied dc voltage to a conductive cantilever-probe ensemble (Fig. 6A), one may, for example, discern buried CNTs distributed in a poly(styrene-b-ethylene butylene-b-styrene) (SEBS) polymers, as shown by Thompson *et al*. (*81*) This method can be considered a modality of electrostatic FM (EFM), typically invoked for polymer composite characterization (*82*). EFM is performed in two steps beginning with the standard tapping-mode imaging to acquire topography, followed by adjusting the tip height to sense the long-range tip-sample forces and capture subsurface features. EFM has enabled the imaging of single-walled CNTs (SWCNTs) in a polymer matrix (Fig. 6B) (*83*). To simultaneously obtain the topography of the polyimide matrix and the image of the embedded SWCNTs, Cadena *et al*. (*84*) proposed a single-step EFM, where the conductive cantilever is vibrated at the free resonance frequency of the cantilever *f*_0_, while an ac voltage with a frequency *f_v_* < *f*_0_ is applied to the cantilever. Using lock-in detection, the topography is mapped, while the phase of the cantilever signal is measured with reference to (*f_v_*). Recently, the contact EFM (*85*), based on the combination of contact-mode AFM and EFM, was proposed to detect the charges hidden under atomic 2D crystals. In this method, the tip maintains contact with the sample surface, while a combination of dc and ac voltages is applied to the back-gated substrate and the bending of the grounded cantilever is measured.

To study the dependence of the lateral resolution upon the depth of the embedded materials, Castañeda-Uribe *et al*. (*86*) used a single-step EFM and kelvin probe FM (KPFM). The KPFM measures the contact potential difference between a conducting AFM tip and a sample. By approaching the AFM tip toward the sample surface, reliant on a feedback-based nullification of long-range electrostatic forces, the KPFM extracts the fundamental electronic properties via the simultaneous application of ac and dc signals between the sample and the tip. Castañeda-Uribe *et al*. (*86*) used the second harmonic of the KPFM signal to analyze the depth sensitivity of polymer nanocomposites, demonstrating the ability to identify the local capacitance gradient over the sample and at the interfaces of nanomaterials and polymers. A comparative study of dc-biased AM-AFM, single-pass EFM, and KPFM (*86*, *87*) concluded that the KPFM is the most robust technique with good stability against contaminations and surface charges (see table S1).

Increasing interest in magnetic drug delivery of materials and labeled cells has brought attention to the development of magnetic FM (MFM) for subsurface visualization (*88*). In MFM, the detection of a signal that originates from magnetic structures buried in nano/biomaterials provides a channel for magnetic imaging, which is co-registered with the topography. In MFM, contact or tapping-mode AFM operates in conjunction with the signal containing magnetic information. The magnetic signal component uses both static (deflection), and dynamic (frequency and/or phase shift) behavior of the cantilever (*87*, *88*). The most common mode is the resonant MFM which provides higher sensitivity and less damage to the sample during the imaging (*87*). Like the two-step EFM, the resonant MFM is also implemented in two steps. First, tapping-mode AFM is invoked to acquire the topography, and then the cantilever is lifted to a constant height and oscillated at its free resonance frequency *f*_0_. Because of the magnetic coating of the cantilever tip and the magnetic structures distributed at the surface and subsurface of the sample, the cantilever experiences a force during sample scanning, which can be detected and used to provide the magnetic images of the sample. Nocera *et al*. (*89*) used the MFM to detect the Fe core buried in the ferritin matrix. Similarly, MFM was used to detect Fe core embedded in magneto ferritin matrix (*87*), ferritin buried in spleen tissue (*90*), and iron oxide NPs buried in A375M and MCF7 cells, respectively (*91*). Moreover, Dong *et al.* (*92*) verified the encapsulation of magnetic NPs inside the noisome (Fig. 6C).

Scanning microwave microscopy (SMM)—an SPM technique of potential for subsurface studies—allows high lateral and depth resolution measurements of the electrical and magnetic properties of nanostructures buried in the nanomaterials (*88*, *93*–*101*). In SMM, microwaves are emitted by a cantilever tip through a sample having a complex impedance, and the reflected and transmitted signals are detected. The involved impedance receives contributions from the surface at the contact point of the tip and from the subsurface of the sample. SMM modes include contact, lift, constant height, and intermittent contact (*94*). In contact mode, the tip is continuously in contact with the specimen, while, in intermittent contact mode, the tip is tapped as in tapping-mode AFM (*97*). In the constant height mode, the capacitance is measured at a constant distance from the substrate, while, in the lift mode, the height is controlled to keep a constant probe-sample distance. Using SMM, Biagi *et al*. (*98*) reported intrinsic capacitance images from which dielectric nanorods were detected 150-nm deep in bacterial cells. Using SMM, Tselev *et al*. (*99*) detected PS particles in glycerol packed under a 50-nm-thick SiN membrane (Fig. 6D), Ag electrodes on the backside of the dielectric membrane in water, and yeast cells immersed in glycerol under an 8-nm-thick SiO_2_ membrane. The capacitance was acquired following a sensitivity calibration (1.6 aF/mV) of the microscope. SMM transmission mode has allowed imaging of the dopant concentration in a thick layer of the silicon substrate (*102*). Plassard *et al*. (*96*) imaged aluminum structures 95 nm under a layer of Ni using SMM contact mode. Recently, Gramse *et al*. (*93*) used SMM to image the patterns and conductivity of phosphorous layers embedded in Si(100) wafers (Fig. 6E). You *et al.* (*100*) used the reflected amplitude and phase of the SMM signal to image metal lines buried 800 nm in the plasma-enhanced chemical vapor deposition–tetraethylorthosilicate (PECVD-TEOS) dielectric layer (with a maximum detectable depth of 2300 nm). The high lateral resolution of SMM provides the opportunity to image interfaces, doped regions, and junctions of semiconductors (Fig. 6F) (*101*). In Fig. 6F, in the areas where the concentration of doping is higher, the capacitance map displays higher values. The images of reflected topography, and calibrated capacitance of a flat p-n junction structure, are shown in Fig. 6G (*103*).

## THERMAL-BASED METHODS

**Fig. 7.**
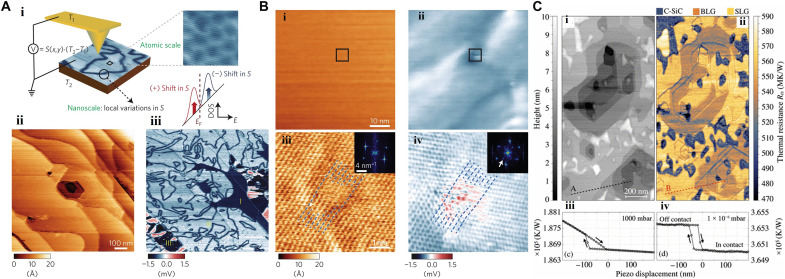
SPM-based thermal excitation. (**A**) (i) The contact of a conductive probe with a sample surface leads to a heat flow from the tip to the sample inducing a thermoelectric voltage, the measurement of which enables thermal mapping of the sample. (ii and iii) Co-registered AFM topographic height (ii) and thermopower (iii) images of epitaxial graphene [adapted with permission from Cho *et al*. (*108*)]. (**B**) Thermoelectric identification of a local defect in bilayer epitaxial graphene: (i) topography, (ii) co-registered thermoelectric map, (iii) topography of the area indicated by a square in (i), and (iv) thermoelectric image of the area indicated by a square in (ii) [adapted with permission from Cho *et al*. (*108*)]. (**C**) Thermal resistance of graphene on SiC: (i) the topography of the graphitized SiC surface corresponding to (ii) the thermal resistance map with the SiC buffer layer (C-SiC), bilayer graphene (BLG), and single-layer graphene (SLG) (iii) probe-specimen approach curve under either ambient pressure or (iv) high-vacuum conditions [adapted with permission from Menges *et al*. (*111*)].

Thermal properties of specimens may be exploited for subsurface studies (see “Thermal state” in Fig. 1). In scanning thermal microscopy (SThM), a nanoprobe, behaving as a thermometer and a resistive heater, enables the mapping of thermal properties of both surface and subsurface materials including thermal conductivity and phase transition (*104*–*107*). SThM operates in both the contact and noncontact modes (*106*). Using the contact mode, Mills *et al*. (*105*) studied voids in a SiO_2_ passivation layer. Cho *et al*. (*108*), using SThM (ultrahigh vacuum AFM with a gold-coated conductive cantilever operated in contact mode), uncovered point defects in the first layer of epitaxial graphene (Fig. 7A). The thermoelectric voltage proportional to the local thermopower of the contact area is obtained because of the localized temperature gradient induced in the vicinity of the probe. Local variations in the density of states (DOS) in the vicinity of the Fermi level, such as additional DOS from defects, lead to local changes in thermopower. This can be detected and used as a contrast for mapping the topography. The thermoelectric voltage and the vertical displacement of the cantilever may be recorded simultaneously, allowing the topographic and thermoelectric DOS-induced features to be distinguished. Exploiting this approach, the nanoscale line patterns and spots are imaged (Fig. 7B, i and ii), while the intersecting line patterns are illustrated in the close-up images shown in Fig. 7B (iii and iv). The complex interference pattern is explored from a thermoelectric image (Fig. 7Biv), which originates from electron scattering. The inset of Fig. 7Biv, exhibiting the Fourier transform of this scattering image, proposes that electrons are scattered with the wave vector very close to the Fermi wave vector (*109*). This provides evidence on thermopower imaging to extract information on the DOS. SThM has further allowed mapping the local Peltier effects at the metal-semiconductor contacts to an indium arsenide nanowire (*110*), and the local transfer of heat to graphene on amorphous SiO_2_ and crystalline silicon carbide (SiC) (Fig. 7C) (*111*).

Scanning near-field thermoelectric microscopy (SteM) is a member of the SThM family with remarkable applicability to quantifying the thermoelectric properties of nanomaterials (*112*). In SteM, a thermal probe contacts the surface of a thermoelectric specimen. By heat-modulating the probe, near-field evanescent thermal waves are induced throughout the contact region. This leads to a thermoelectric near-field interaction and consequent excitation of three harmonic signals. Then, the local Seebeck coefficient can be determined from the slope of the second harmonic voltage plotted against the ratio of the third harmonic to the first harmonic voltage (*112*). Therefore, the Seebeck voltage at different thermal penetration depths can be determined and used to explore the subsurface thermoelectric properties of materials.

Using SteM, Xu *et al*. (*112*) characterized the thermoelectric properties of the Ag_2_Se and studied the dependence of the Seebeck voltage of Ag_2_Se on the temperature at different modulation frequencies and the frequency dependence of the Seebeck coefficient.

Scanning thermal noise microscopy (STNM) is another class of SThM in which the thermal noise of the cantilever is exploited (*113*). By collecting the CR spectrum of a thermal noise–driven cantilever, Yao *et al.* (*114*) imaged the featureless topography of a photopolymer specimen and the noise magnitude of gold NPs buried at a depth of 300 nm.

## FORCE-VOLUME METHOD

The simple yet powerful force-volume method is based on the analysis of the force-distance curves (FDCs) describing the tip-sample interaction as a function of their separation distance (see “Interaction force model” in Fig. 1). The height, surface energy, and deformation of a sample can be quantified by allowing studies of the elastic moduli and energy dissipation of specimen at different indentations. In FDC-based AFM, recording the curve at each point of a specimen enables the nanomechanical and viscoelastic properties of materials to be determined, as demonstrated in cell studies (*115*, *116*).

**Fig. 8.**
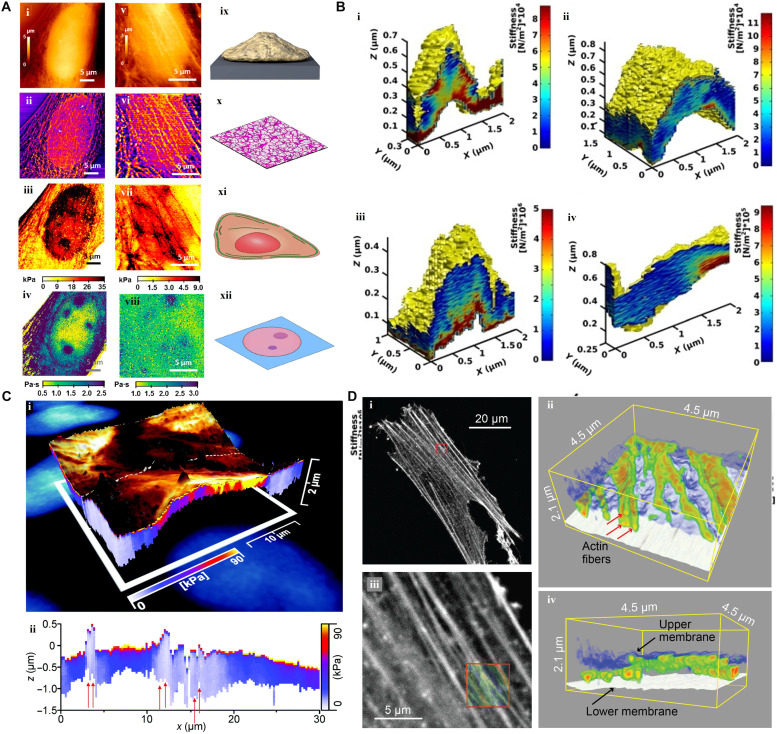
SPM-based force-distance approach for subsurface imaging. (**A**) Viscoelastic mapping of fixed and live NIH-3 T3 fibroblast cells: (i) topography of fixed cell, (ii) topography of actin cytoskeleton with cell depth between 0 and 500 nm, (iii) stiffness of cell with cell depth of 500 nm, (iv) viscous coefficient of the cell with cell depth of 1000 nm, (v) surface topography of live cell, (vi) topography of actin cytoskeleton with live cell depth between 0 and 100 nm, (vii) stiffness of cell at 500-nm depth, (viii) viscous coefficient of the live cell at 1000-nm depth, (ix) schematic of a single cell on a substrate, (x) actin-based cell cortex schematic, (xi) schematic of cell elements with substantial incorporation in cell stiffness, and (xii) cell organelles immersed in the nucleosol schematic [adapted with permission from Guerrero *et al*. (*7*)]. (**B**) Stiffness tomography of four different living neurons [adapted with permission from Roduit *et al*. (*117*)]. (**C**) (i) Volume image of the cancerous epithelial breast cell displaying surface roughness and nanomechanical properties. (ii) A vertical slice along the dashed line in (i) [adapted with permission from Stühn *et al*. (*118*)]. (**D**) Confocal microscopy and 3D nanoendoscopy-AFM mapping the fluorescence (i) from the stained actin filaments, amplified in (ii) for the red square region in (i). Cytoskeleton actin fibers (iii and iv) were recorded for the red square region in (ii). The superimposed image in the red square in (ii) is the projection of the 3D images of (iii) and (iv) [adapted with permission from Penedo *et al.* (*122*)].

Guerrero *et al*. (*7*) proposed a subsurface technique for imaging cellular organelles based on a classification of the FDCs at different indentation sections (Fig. 8A). Figure 8A shows the topography and stiffness maps of a fixed (Fig. 8A, i to iv) and a live fibroblast cell (Fig. 8A, v to viii), respectively (*7*). Using FDCs, Roduit *et al*. (*117*) performed stiffness tomography of four different living neurons, as seen in Fig. 8B, where the red areas show the cortical actin cytoskeleton under the cell membrane. Figure 8C shows the 3D image of local and cumulative nanomechanical properties of cancerous epithelial breast cell (*118*). The changes of *z* piezo at the contact point of each set of FDCs represent the membrane roughness. As seen in Fig. 8Cii, three different nuclei can be recognized from a color map of stiffness which would not be possible using the membrane image. The FDC methods have been also applied to quantify the stiffness changes in fixed and living macrophages (*119*), bacterial membranes (*120*), and vegetal cells (*121*). Recently, Penedo *et al*. (*122*) proposed nanoendoscopy-AFM in which a nanoprobe repeatedly indents a cell’s interior to collect FDCs. Both 3D maps of actin fiber (Fig. 8D) and 2D nanodynamics of the membrane inner scaffold were explored.

## OUTLOOK

The reviewed work on subsurface studies suggests that advances made in high-resolution visualization of the internal structures of materials have been primarily in the ranges of 1 to 400 nm, laterally, and in 2 nm to 8 μm, vertically. Whereas these metrology modalities have been relatively successful in the investigation of the forward problem, major bottlenecks remain in the case of the inverse problem. Thus, even if an unknown object can be detected by, e.g., the reviewed frequency mixing techniques, determining its properties quantitatively remains a major challenge. As described, via the mechanical, thermal, or electromagnetic approach, it is possible to detect the subsurface object and roughly quantify it, if a calibration scheme can be devised. However, devising calibration schemes for subsurface signal transductions is an involved and challenging undertaking in and of itself. Nanofabrication methods could be invoked to design reasonably well-defined shapes, material constituency, and spatial distribution (depth placement). Nonetheless, the full set of properties of a given material requires the integration of different methods in a standard platform and/or combination with complementary microscopy techniques as well as using artificial intelligence to associate explored properties with a library of materials. To provide a framework for the quantification of materials properties at both surface and subsurface levels, the integration of nanoscale instrumentation with advances in data analysis, modeling, and computational methodologies is required. In light of the availability of higher computing power, recent achievements in data analysis and quantification for AFM methods as well as advances in Bayesian, deep learning, and signal and image processing techniques are opening advanced possibilities (*123*–*125*), which can be extended to subsurface techniques. Without high measurement speeds, it is unlikely to detect fast dynamics phenomena including charge transfer, phonon propagation, elucidation of donor-acceptor phase separation, and thermal and conductive gradient inside the materials. For biological applications, real-time visualization of subcellular dynamics and morphology of living cells with nanometer resolution remains challenging. These properties can be revealed through in situ measurement which needs notable development in the time-resolution operation of SPM techniques. Recent development in high-speed AFM measurement (*126*) can be explored for adaptation in subsurface imaging. Any nonlinearity and inhomogeneities in the sample surface region, in addition to those of the subsurface, can notably alter the measurements. Transient dynamics, the complexity of probe-sample interaction, and the lack of universal and inclusive tip-sample contact mechanics models (besides artifacts that could come from the atomic scale morphology of the probe) make the data acquisition and analysis of SPM subsurface methods complex and time-consuming. A deeper understanding of and model for contact mechanics is also urgently needed for the characteristics of the attachment of the buried materials to the host. The role of friction and local dissipation at the buried-host interface is of explicit relevance to an understanding of the subsurface signal formation. Recent works on data acquisition reported for surface characterization and imaging can be applied and developed for subsurface application (*127*, *128*). Other remaining challenges in subsurface methods include the complex implementation and equipment costs which yield unviable commercial calculations. Moreover, because of more complexity of the cantilever-surface and subsurface interaction in a liquid environment, implementation of SPM techniques in a liquid is considerably more challenging than in an air environment. Despite this, recent methods of FDC, SMM, SNFUH, and MSAFM show the potential to image and characterize the subcellular structure. These in-liquid subsurface methods are still amenable to enhancement for in vivo measurements. The in-liquid operation would be a tremendous capability for the study of the live specimen but presents added challenges due to fluid coupling. Thickness variations can also affect the results, like the bottom (substrate) effect in indentation studies for quantitative elasticity measurements. While still in its infancy, quantum sensing is emerging as the next-generation nanometrology. Powerful measurement channels are being explored on the basis of either the quantum states of the probe—most notably mechanical state—or the readout and soon, as may be expected, both (see “Quantum state” in Fig. 1). Quantum nanomechanical squeezed states (*129*) and probe state readout using entangled and squeezed state photons (*130*) are drawing increasing attention to enhance measurement sensitivity and limit of detection via noise suppression and unique quantum correlations. Topological materials offer tantalizing opportunities in quantum subsurface studies. For example, skyrmions—spin excitations in certain magnetic materials—may be manipulated by a scanning tip over the surface where a current can be injected, and subsurface information may be collected. The subsurface problem viewed as an inverse problem encompassing many-body interactions could benefit from, for example, real-time density functional theoretic tip-surface models and machine learning algorithms computed on a quantum computer. Although these topics seem far-fetched, the reviewed nanometrology work is preparing the ground for their exploration.

## Supplementary Materials

### This PDF file includes:

Tables S1 and S2 References
