# Supplementary material for: Imaging beyond the surface region: Probing hidden materials via atomic force microscopy
Source: Sci Adv. 2023 Jun 28;9(26):eadg8292. doi: 10.1126/sciadv.adg8292 (PMC10306303; doi:10.1126/sciadv.adg8292)
Supplement: Supplementary file 1 — Tables S1 and S2 References [file sciadv.adg8292_sm.pdf]

Science Advances
AAAS

Supplementary Materials for

Imaging beyond the surface region: Probing hidden materials via atomic force microscopy

Amir Farokh Payam and Ali Passian

Corresponding author: Amir Farokh Payam, a.farokh-payam@ulster.ac.uk; Ali Passian, passianan@ornl.gov

Sci. Adv. **9**, eadg8292 (2023)
DOI: 10.1126/sciadv.adg8292

This PDF file includes:

Tables S1 and S2
References

Table S1.
Subsurface technology matrix.

Nanometrology			Implementation & Merit			
Technology & Target study			Transduction	Data	Strength & Weakness	
**Measurement Modality**	**Probe features & stiffness *k* (N/m)**	**Specimen**			**Pros**	**Cons**
AM-AFM(*9*)	*k*=16.5-21.2	Polymers; Soft matrix	Amplitude ✓<br>Phase ✓<br>Indentation ✓<br>Frequency ✗	Quantitative ✗<br>Topography ✓<br>Subsurface property ✗	Depth resolution; Eliminating the effect of lateral force; No extra LIAs; Ambient condition	Effect of tip radius/shape; Indentation dependence; Lack of subsurface properties
Bi/tri-modal AFM(*42*, *43*)	*k*=2.3-2.7	Silicon nanowires & Fe_2O_3 under films; Glass NPs under PDMS	Amplitude ✓<br>Phase ✓<br>Indentation ✓<br>Frequency ✗	Quantitative ✗<br>Topography ✓<br>Subsurface property ✗	High sensitivity; Depth resolution; Extra imaging channels; High scan rate; Ambient condition	Need extra LIAs (two/three); Indentation dependence; Lack of subsurface properties
Multi-harmonic AFM(*47*)	15 μm tip height, 50-300 nm radius; diamond-like tip; *k*=0.9	Subcellular structure	Amplitude ✓<br>Phase ✓<br>Indentation ✓<br>Frequency ✗	Quantitative ✓<br>Topography ✓<br>Subsurface property ✓	High spatial resolution; Minimal topography artefacts; High penetration depth; Nanomechanical properties	Need special cantilevers; Only mechanical properties quantification; Slow scan speed (5-10 mins per image)
UAFM(*39*, *40*)	*k*=0.19-16	Nanoparticles in Polymaide, silicon, and metals; Soft/hard matrices; Defects of HOPG	Amplitude ✓<br>Phase ✓<br>Indentation ✓<br>Frequency ✗	Quantitative ✓<br>Topography ✓<br>Subsurface property ✓	Sample or cantilever excitation; Operation in both linear/nonlinear regime; Easy to use	Only mechanical properties quantification
FMM(*40*, *50*, *72*)	*k* =0.01-1.3	Nanoparticles in polymers; Polymers inclusion; Generally soft samples	Amplitude ✓<br>Phase ✓<br>Indentation ✓<br>Frequency ✗	Quantitative ✗<br>Topography ✓<br>Subsurface property ✗	Less force applied to the sample; Depth resolution; Ambient condition; Localized sample excitation	Tip/sample damage; Low cantilever spring constant; Friction effect; Low sensitivity; Lack of subsurface properties
AFAM(*40*, *50*, *54*–*56*)	*k* =0.1-14	Detect nanoparticles, defects, and subsurface features in a diverse range of materials	Amplitude ✓<br>Phase ✓<br>Indentation ✓<br>Frequency ✗	Quantitative ✗<br>Topography ✓<br>Subsurface property ✗	Decrease friction in high frequency vibration; Applicable to diverse range of materials; Diverse range of cantilevers; Ultrasonic imaging; Deeper imaging with nanoscale resolution; Minimize topography artefacts; Localized sample excitation	Qualitative; Low scan speed; Time consuming; Lack of subsurface properties
UFM(*48*, *60*–*62*, *72*)	*k* =0.09-1	Detection nanoparticles, defects and subsurface	Amplitude ✓<br>Phase ✓	Quantitative ✗<br>Topography ✓	Very effective technique to detect variations in the stiffness of	The sample contact stiffness should be much larger than cantilever stiffness;

		features in diverse range of materials	Indentation ✓<br>Frequency ✗	Subsurface property ✗	stiff samples using a soft cantilever; Depth resolution; Localized sample excitation	Qualitative; Low scan speed; time consuming; Lack of subsurface properties
CR-AFM(*63–65*, *67*)	k =0.11-2.58	Diverse range of materials	Amplitude ✗<br>Phase ✗<br>Indentation ✓<br>Frequency ✓	Quantitative ✓<br>Topography ✓<br>Subsurface property ✓	Provide stiffness image; Localized sample excitation; Easy implementation without need to special cantilever and sample; soft cantilevers give higher subsurface contrast	Affected by shape/size of the tip; limited resolution; Required large applied force and lower sample thickness to improve resolution; Effect of topographic artifacts; limited for small size nanoparticles; Imaging noise for softer cantilevers
HFM(*39*, *40*, *72*)	k =0.16-1.3	Detect subsurface defects and nanoparticles inside polymers and HOPG	Amplitude ✓<br>Phase ✓<br>Indentation ✓<br>Frequency ✗	Quantitative ✗<br>Topography ✓<br>Subsurface property ✗	Tip and sample are oscillated at much higher frequencies; Significantly decrease friction; Depth resolution; Using the information of relative phase between two ultrasound excitations	Qualitative; Lack of subsurface properties; Implementation complexity; Need extra hardware including mixer and function generators; The contrast is affected by size of the characteristic features with respect to the wavelength, tip-sample interaction and friction
ICR-AFM(*75*)	k =8.1	Inhomogeneous superficial layers, either inorganic or organic materials	Amplitude ✗<br>Phase ✗<br>Indentation ✓<br>Frequency ✓	Quantitative ✓<br>Topography ✓<br>Subsurface property ✓	Depth resolution; 3D tomographic data; High scan speed; Localized sample excitation	Need extra hardware to excite both sample and cantilever; Effect of tip shape/size; Iterative computation
MSAFM(*14*, *77–79*, *131*)	k =0.06-42	Diverse range of materials	Amplitude ✓<br>Phase ✓<br>Indentation ✓<br>Frequency ✗	Quantitative ✗<br>Topography ✓<br>Subsurface property ✗	Possibility to extract more data from measurement by frequency couplings; Diverse range of cantilevers; Localized sample excitation; Depth resolution	Complex implementation; Need extra LIAs; Nature of image contrast is not clearly known; Optimal choice of parameters is challenging; Qualitative
SNFUH(*26*, *27*, *73*, *74*, *132*)	N.A.	Diverse range of materials	Amplitude ✓<br>Phase ✓<br>Indentation ✓<br>Frequency ✗	Quantitative ✗<br>Topography ✓<br>Subsurface property ✗	High penetration depth; Depth resolution; No limit in actuation frequency; Localized sample excitation	Qualitative; Effect of materials properties, geometry and frequency selection is unknown; Hardware complexity
DC-AFM(*81*, *84*)	High aspect ratio (HAR) probes;<br>k =1.3-2.8	Polymers; Composites	Amplitude ✓<br>Phase ✓<br>Indentation ✗<br>Frequency ✗	Quantitative ✓<br>Topography ✓<br>Subsurface property ✓	High scan speed; Ambient condition; Measured dissipation; Easy implementation (one LIA); Detect high dielectric constant materials in low dielectric constant matrix	Depth range is limited by electric field penetration to the sample; Required high voltage for thick/nonconductive samples; Topography artefacts; Presence of noise; Operated just in attractive regime
EFM(*27*, *83*)	Conductive cantilevers;<br>k =0.2-5.4	Polyamide composite; Polymers; SiO_2;	Amplitude ✓<br>Phase ✓	Quantitative ✓<br>Topography ✓	Measure electrostatic force; Ambient condition; High	Need extra LIA; Limited to electrostatic

		SWNT; 2D materials	Indentation ✗<br>Frequency ✗	Subsurface property ✓	contrast; No feedback for AC signal	properties; Slow scan speed
KPFM(*84*)-(*86*)	Conductive cantilevers; *k* =0.5-4.4	Polyamide composite; Polymers; SiO_2; SWNT; 2D materials	Amplitude ✓<br>Phase ✓<br>Indentation ✗<br>Frequency ✗	Quantitative ✓<br>Topography ✓<br>Subsurface property ✓	Surface potential and capacitance gradient; High contrast; Robust and accurate	Need extra LIA (two/three); Required tunning two feedback loops; Slow scan speed; Influence of surface morphology on surface potential; Limited to electrostatic properties
MFM(*88*)-(*92*)-(*133*)-(*134*)	Magnetic coated cantilevers; *k* =1-5	Metals and magnetic nanoparticles in Ferritin; Magneto ferritin; Biological cells; Spleen	Amplitude ✓<br>Phase ✓<br>Indentation ✗<br>Frequency ✗	Quantitative ✗<br>Topography ✓<br>Subsurface property ✓	High sensitivity and spatial resolution; Topography and MFM amplitude/phase imaging; Ambient condition; Easy to operate	Qualitative; Magnetic properties only; Challenges in modelling tip-surface interaction; Challenges to quantify magnetic properties; Low scan speed
SMM(*93*)-(*102*)-(*135*)-(*136*)	Platinum coated cantilevers; Shielded tip; *k* =0.3-18	Defects, voids, nanoparticles, subcellular structures; Diverse types of materials (e.g., polymers, cells, semiconductors, bacteria)	Amplitude ✓<br>Phase ✓<br>Indentation ✗<br>Frequency ✗	Quantitative ✓<br>Topography ✓<br>Subsurface property ✓	Capacitance; Resistance; High penetration depths; High contrast; High resolution; Localized sample measurements; High penetration depths; Identification of dielectric and metallic reaction products	Expensive, need extra hardware (e.g., VNA, LIA); Challenging in liquid environment; Affected by thickness and tip radius; Need modelling the effect of transmission line and changes in resistive and reactive components of tip-sample impedance; Frequency dependence of skin depth and penetration depth
SThM(*105*, *108*, *110*, *111*)	Gold/diamond coated cantilevers; *k* =0.15-0.5	Diverse types of materials	Amplitude ✗<br>Phase ✗<br>Indentation ✗<br>Frequency ✗	Quantitative ✓<br>Topography ✓<br>Subsurface property ✓	Measure topography in contact mode; Thermal resistance; Thermoelectric imaging; High resolution; Depth resolution; Quantification of the density of state	Limited range of energy bands; Susceptible to variations (e.g., due to topography/materials heterogeneity); Affected by contact size and tip-sample contact mechanics
STeM(*112*)	Thermal resistance probe	Thermoelectric materials	Amplitude ✓<br>Phase ✗<br>Indentation ✗<br>Frequency ✗	Quantitative ✓<br>Topography ✗<br>Subsurface property ✓	Using harmonics signal to measure nanoscale Seebeck coefficient of thermoelectric materials; High lateral resolution; Depth sensitivity	Limited to thermal probe and thermoelectric materials
STNM(*114*)	Al-coated Si cantilevers; *k* =1.2	Photopolymer films with buried Au nanoparticles	Amplitude ✓<br>Phase ✗<br>Indentation ✗<br>Frequency ✓	Quantitative ✗<br>Topography ✓<br>Subsurface property ✗	Thermal noise spectrum; No extra hardware; Easy to use; Depth resolution	Fitting based approach; Significant dependence on depth; Lack of subsurface properties material properties; Qualitative; Cantilever damping effects

FDC(*7*, *117–122*)	*k* =0.06-0.3	Subcellular structures	Amplitude ✗<br>Phase ✗<br>Indentation ☑<br>Frequency ✗	Quantitative ☑<br>Topography ☑<br>Subsurface property ☑	Straightforward, force curve-based; Adhesion, viscoelasticity, and stiffness; High penetration depths	Tip-indentation, Strongly depends on stiffness of samples; Time consuming; Requires contact mechanics of cells and biological samples

Table S2.

Overview of the different nanomaterials/sample combinations, techniques, sample thickness, materials size, and detection depth. C = cantilever, S = sample.

Method	Excitation	Sample	Sample thickness	Subsurface entities	Entity size	Depth penetration	Ref.
**AM-AFM**	C	Polybutadiene (PB) matrix	60 nm	PS Block copolymer	NA	20 nm	(*9*)
**AM-AFM**	C	Polybutadiene (PB) matrix	240 nm	Semi-crystalline polymer	10 nm	20 nm	(*9*)
**DC-biased AFM**	C	Polyamide	40-100 nm	SWCNT/SEBS composite	NA	<100 nm	(*81*)
**DC-biased AFM**	C	Poly(styrene- b-ethylene butylene-b-styrene) (SEBS) polymer blend	40-100 nm	DWCNT/SEBS composite	NA	<100 nm	(*81*)
**DC-biased AFM**	C	SWCNT/Polyimide composite	25~35 μm	SWCNT	1.5 nm (D)	NA	(*84*)
**EFM**	C	SWCNT/Polyamide composite	25-65 μm	SWCNT	1-3 nm (D)	<1.6 μm	(*82*)
**EFM**	C	SWCNT/PMMA composite	170 nm	SWCNT	1-3 nm (D)	50-60 nm	(*83*)
**EFM**	C	Polymeric film	430 $\pm 50\ nm$	domains of a methyl-terminated monolayer surrounded by domains of an alcohol-terminated monolayer	NA	NA	(*137*)
**C-EFM**	S	SiO_2/ doped Si substrates	NA	Few layers graphene and MoS2	5.4 nm	NA	(*138*)
**EFM**	C	SWCNT/Polyimide composite	25~35 μm	SWCNT	1.5 nm (D)	NA	(*84*)
**KPFM**	C	SWCNT/Polyimide composite	25~35 μm	SWCNT	1.5 nm (D)	NA	(*84*)
**KPFM**	C	SWCNT/Polyimide composite	20 μm	SWNT	1-3 nm (D)	430 nm	(*86*)
**KPFM**	C	Polyaniline-polystyrene film, polystyrene film	80-100 nm	Au	NA	NA	(*139*)
**MFM**	C	Ferritin	12 nm (D)	Fe core	8 nm (D)	2 nm	(*89*)
**MFM**	C	Magnetoferritin	12 nm (D)	Fe core	8 nm (D)	2 nm	(*88*)
**MFM**	C	Spleen tissue	NA	Ferritin	12 nm	NA	(*90*)
**MFM**	C	Microgelia cell	NA	Fe_3O_4 NPs agglomerates	4 μm	NA (WC)	(*64*)
**MFM**	C	Niosome (Span 20)	NA	Magnetic NPs	50 nm	150 nm	(*92*)
**MFM**	C	Niosome (Tween 20)	NA	Magnetic NPs	20 nm	200 nm	(*88*)
**MFM**	C	MCF7 cells	NA	$\gamma - Fe_2O_3$ NPs	30 nm	NA (WC)	(*133*)
**MFM**	C	A375M cells	NA	Iron oxide NPs	NA	NA	(*91*)
**MFM**	C	MCF7 cells	NA	Iron oxide NPs	NA	NA (WC)	(*91*)
**MFM**	C	HL-60 cells	NA	SiO_2-coated iron NPs	NA	NA (WC)	(*134*)
**FMM**	S	Polystyrene	500 nm	Cobalt NPs	25 nm	NA	(*50*)
**FMM**	S	Polyimide	200 nm	Gold NPs	50 nm	NA	(*40*)
**FMM**	S	PMMA	3 mm	PMMA/rubber/PMMA inclusions	300 nm (biggest diameter)	NA	(*72*)
**AFAM**	S	PS films	200 nm	Au grating	100 nm	200 nm	(*53*)

**AFAM**	S	PMMA	7 μm	Au lines	50 nm	7 μm	(*53*)
**AFAM**	S	Silicon Cap	NA	L-shaped Al	50 nm	200 nm	(*53*)
**AFAM**	S	Polyimide	900 nm	Au NPs	50 nm	NA	(*40*)
**AFAM**	S	Amorphous carbon film	100 nm	defects	NA	100 nm	(*54*)
**AFAM**	S	MDA-MB-231 breast cancer cells	NA	Subcellular structures	NA (WC)	NA (WC)	(*55*)
**AFAM**	S	MCF7 breast cancer cells	NA	Subcellular structures	90 μm	NA (WC)	(*55*)
**AFAM**	S	Escherichia coli (E. coli)	NA	Subcellular structures	2-4 μm	NA (WC)	(*55*)
**AFAM**	S	Staphylococcus aureus (S. aureus)	NA	Subcellular structures	0.2-0.5 μm	NA (WC)	(*55*)
**AFAM**	S	Onion epidermis	NA	Subcellular structures	16 μm	NA (WC)	(*55*)
**AFAM**	S	Human erythrocytes	NA	Subcellular structures	8 μm	NA (WC)	(*55*)
**AFAM**	S	Cell	3μm	Nucleus, Cytoplasm, Cytomembrane	NA (WC)	NA (WC)	(*56*)
**AFAM**	S	HOPG	18.6-77.1 nm	defects	NA	NA	(*39*)
**AFAM**	S	PMMA photo-resist layer.	7 μm	Gold lines	50 nm	NA	(*53*)
**AFAM**	S	Polystyrene	200 nm	Au NPs	100 nm	NA	(*53*)
**UFM**	S	HOPG	NA	Lattice defects	NA	3 nm	(*60*)
**UFM**	S	SiO_2/ doped Si substrates	NA	Few layers graphene and MoS2	5.4 nm	NA	(*138*)
**UFM**	S	Glass/PET composite	NA	Cracking and debonding	NA	NA	(*140*)
**UFM**	S	Cyclic olefin copolymer (COC) patterned substrate	NA	Graphite	50 nm	NA	(*57*)
**UFM**	S	Cyclic olefin copolymer (COC) patterned substrate	NA	Molybdenum disulphide	15 nm	NA	(*57*)
**UFM**	S	Poly-L-Lysine (PLL) surface	NA	Amyloid-β peptides	1.52, .59, 0.74, 6 nm	NA	(*48*)
**UFM**	S	Photoresist	300 nm	Aluminum	50 nm	NA	(*61*)
**UFM**	S	Photoresist + Titanium layer	300 nm	Aluminum	50 nm	NA	(*61*)
**UFM**	S	SiO_2	300 nm	Aluminum	50 nm	NA	(*61*)
**UFM**	S	PMMA photoresist layer + titanium layer	300 nm+ 50 nm	Aluminum	150 nm	NA	(*62*)
**UFM**	S	PMMA	90 nm	Silicon	50 nm	NA	(*62*)
**UFM**	S	PMMA	3 mm	PMMA/rubber/PMMA inclusions	300 nm (biggest diameter)	NA	(*72*)
**LUFM**	S, C	HOPG	NA	Lattice defects	NA	3 nm	(*60*)
**UAFM**	C	HOPG	18.6-77.1 nm	defects	NA	NA	(*39*)
**UAFM**	S	Polyimide	900, 730, 200 nm	Gold NPs	50 nm	NA	(*40*)
**2nd harmonic UAFM**	S	Polyimide	900 nm	Gold NPs	50 nm	NA	(*40*)
**Trimodal AFM**	C	PDMS film	70 nm	Si nanowires	1.5-2 nm	NA	(*43*)
**Trimodal AFM**	C	PDMS film	65 nm	Fe_2O_3 NPs	7-10 nm	NA	(*43*)

**Trimodal AFM**	C	PDMS	NA	Glass NPs	13.7 nm	30 nm	(*42*)
**Multi-Harmonic AFM**	C	Living NIH 3T3 fibroblasts	4 μm	Subcellular structure	NA	3 μm	(*47*)
**Multi-Harmonic AFM**	C	Breast cancer MDA-MB-231 cells	7.5 μm	Subcellular structure	NA	4.3 μm	(*47*)
**HFM**	S, C	PMMA	3 mm	PMMA/rubber/PMMA inclusions	300 nm (biggest diameter)	NA	(*72*)
**HFM**	S, C	Polyimide	960 nm	Gold NPs	50 nm	NA	(*40*)
**HFM**	S, C	HOPG	18.6-77.1 nm	defects	NA	NA	(*39*)
**CR-AFM**	S	PS films	NA	Silica NPs	50 nm	32, 92, 125, 160 nm	(*63*)
**CR-AFM**	S	6H-SiC (0001)	NA	graphene	Few nanometer	NA	(*65*)
**CR-AFM**	S	Microglia cell	NA	Fe_3O_4 NPs	400 nm	100 nm	(*64*)
**CR-AFM**	S	PMMA	52,117,185, 380,653 nm	flexible circuit	NA	NA	(*67*)
**ICR-AFM**	S, C	SiO2 thin film substrate	NA	Hybrid organic/inorganic silicate low-k dielectric	100 nm	NA	(*75*)
**SNFUH**	S, C	PVP	500 nm	Au NPs	15 nm	~ 568-573 nm	(*73*)
**SNFUH**	S, C	Infected red blood cells (RBCs).	NA	Malaria parasites inside RBCs	NA	NA	(*73*)
**SNFUH**	S, C	Benzocyclobutene (BCB) (Cover layer)	500 nm	Si_3N_4 trenches	50 nm	1 μm	(*73*), (*132*)
**SNFUH**	S, C	Mouse cell	NA	Cell nucleus	1 μm	NA	(*74*)
**SNFUH**	S, C	Alveolar macrophages	NA	SWCNH (Single wall Carbon Nanohorn)	70-100 nm	NA	(*26*)
**SNFUH**	S, C	Red Blood Cell	NA	SWCNH (Single wall Carbon Nanohorn)	70-110 nm	NA	(*26*)
**SNFUH**	S, C	Red blood cells	NA	Silica Nanoparticles	95 nm	NA	(*27*)
**SNFUH**	S, C	Soft polymeric material	500 nm	Nanocavity/ Au/CNHs	50 nm	NA	(*141*)
**SNFUH**	S, C	Mo/Si multilayer	280 nm	Buried void pattern/ Au/CNHs	65 nm	NA	(*141*)
**MSAFM**	S, C	Silicon Substrate	NA	PMMA films	300 nm	NA	(*14*)
**MSAFM**	S, C	Red blood cell	NA	Silica NPs	87 nm	NA	(*78*)
**MSAFM**	S, C	Alveolar macrophage	NA	SWCNH	100 nm	NA	(*78*)
**MSAFM**	S, C	Ni	95 nm	Aluminum	114/17 nm	95 nm	(*79*)
**MSAFM**	S, C	Poplar wood	10-50 μm	Layers of cell (secondary cell wall (SCW), the cell corner (CC) and the middle lamella (L))	NA	NA	(*77*)
**MSAFM**	S, C	Cell walls of fresh Populus	10-50 μm	Layers of cell	NA	NA	(*131*)
**HPFM**	S, C	Cell walls of fresh Populus	20 μm	Biomass study at various chemical processing	NA	NA	(*80*)
**SMM**	C	Escherichia coli cells	300 nm	Dielectric nanorod	150 nm	NA	(*98*)
**SMM**	C	SiN membrane	50 nm	Polystyrene particles in glycerol closely	1.5 μm	100 nm	(*99*)

**SMM**	C	SiO2 membrane	8 nm	Saccharomyces cerevisiae yeasts cells immersed in glycerol	NA	Few hundred s nm	(*99*)
**SMM**	C	SiN membrane	50 nm	thin-film silver electrodes	50 nm	NA	(*99*)
**TM-SMM**	C, S	Polymer plate (cover layer) & Substrate	600 nm 300 nm	Disk	200 nm	1 μm	(*102*)
**SMM**	C	Ni (cover layer) & Si (substrate)	95 nm	Aluminum	20 nm	125 nm	(*96*)
**SMM**	C	Plasma deposited silicon dioxide	800 nm to 2.3 μm	Al–Si–Cu metal lines	1.2 μm	900-1200 nm	(*100*)
**SMM**	C	Doped Si (substrate)	NA	SiO_2	100-400 nm	400 nm	(*94*)
**SMM**	C	Semiconductor wafer of Si	50-450 nm	Defects	NA	450 nm	(*94*)
**SMM**	C	THP1 cell	NA	Subsurface structure	NA	300-500 nm	(*135*)
**SMM**	C	Silicon	15 nm	3D Phosphorous	0.2 nm	4-15 nm	(*93*)
**SMM**	C	Silicon dioxide layer	1 nm	Bipolar doped silicon (substrate)	300 nm	NA	(*101*)
**SMM**	C	Chinese hamster ovary cells	1 μm	Subcellular structure	NA	1 μm	(*136*)
**SMM**	C	E. coli bacteria	NA	Subcellular structure	NA	289 nm	(*136*)
**SThM**	C	SiO_2 passivation layer	2 μm	Voids in aluminum wire	NA	1.7 μm	(*105*)
**SThM**	C	SiO_2 passivation layer	2 μm	Voids in aluminum wire	NA	225 nm	(*105*)
**SThM**	C	Plyiamide passivation layer	NA	Cu	0.55 μm	1 μm	(*142*)
**SThM**	C	Epitaxial graphene	NA	Structural disorder	NA	NA	(*108*)
**SThM**	C	Silicon (111) substrate waver coated with amorphous silicon nitride (SiNx)	150 nm	Au	100 nm	NA	(*110*)
**SThM**	C	Silicon substrate covered with silicon oxide	100 nm	InAs wires	120 nm (D)	NA	(*110*)
**SThM**	C	Silicon wafer with silicon oxide (SiO2) substrate	300 nm	Graphene layers coverage	NA	NA	(*111*)
**SThM**	C	Silicon carbide (SiC)(0001) wafer substrate	NA	Graphene layers coverage	NA	NA	(*111*)
**STeM**	C	Ag_2Se thermoelectric bulk	<1 μm	Local subsurface thermoelectric layer		30-300 nm	(*112*)
**STNM**	C	Photopolymer resist	300 nm	Au NPs	40 nm	300 nm	(*114*)
**FDC**	C	NIH-3T3 fibroblast cell	NA	Subcellular structure	NA	1 μm	(*7*)
**FDC**	C	Cancerous epithelial breast cell	NA	Subcellular structure	NA	2.1 μm	(*118*)
**FDC**	C	Living neurons	NA	Cortical actin cytoskeleton	NA	500 nm	(*117*)
**FDC**	C	THP-1 macrophage	NA	Subcellular structure	NA	500 nm	(*119*)
**FDC**	C	Escherichia coli	NA	Subcellular structure	NA	500 nm	(*120*)
**FDC**	C	Arabidopsis thaliana	NA	Subcellular structure	NA	80 nm	(*121*)
**FDC**	C	Cytoskeleton actin fibers	NA	Subcellular structure	NA	2.1 μm	(*122*)
**FDC**	C	HeLa cell	NA	Subcellular structure	NA	8 μm	(*122*)

NA: not assessed, WC: within a cell.

REFERENCES AND NOTES

1. T. Taubner, F. Keilmann, R. Hillenbrand, Nanoscale-resolved subsurface imaging by scattering-type near-field optical microscopy. *Opt. Express* **13**, 8893–8899 (2005).

2. J. Z. Kovacs, K. Andresen, J. R. Pauls, C. P. Garcia, M. Schossig, K. Schulte, W. Bauhofer, Analyzing the quality of carbon nanotube dispersions in polymers using scanning electron microscopy. *Carbon* **45**, 1279–1288 (2007).

3. S. Lee, L. Li, Rapid super-resolution imaging of sub-surface nanostructures beyond diffraction limit by high refractive index microsphere optical nanoscopy. *Opt. Commun.* **334**, 253–257 (2015).

4. A. Hirohata, Y. Yamamoto, B. A. Murphy, A. J. Vick, Non-destructive imaging of buried electronic interfaces using a decelerated scanning electron beam. *Nat. Commun.* **7**, 3–8 (2016).

5. L. Wang, X. G. Xu, Scattering-type scanning near-field optical microscopy with reconstruction of vertical interaction. *Nat. Commun.* **6**, 8973 (2015).

6. A. Raman, S. Trigueros, A. Cartagena, A. P. Z. Stevenson, M. Susilo, E. Nauman, S. A. Contera, Mapping nanomechanical properties of live cells using multi-harmonic atomic force microscopy. *Nat. Nanotechnol.* **6**, 809–814 (2011).

7. C. R. Guerrero, P. D. Garcia, R. Garcia, Subsurface imaging of cell organelles by force microscopy. *ACS Nano* **13**, 9629–9637 (2019).

8. D. J. McClements, H. Xiao, Is nano safe in foods? Establishing the factors impacting the gastrointestinal fate and toxicity of organic and inorganic food-grade nanoparticles. *NPJ Sci. Food* **1**, 6 (2017).

9. E. C. Spitzner, C. Riesch, C. Magerle, Subsurface imaging of soft polymeric materials with nanoscale resolution. *ACS Nano* **5**, 315–320 (2011).

10. W. Zhang, Y. Chen, Y. Hou, W. Wang, H. Liu, L. Zheng, Subsurface imaging of rigid particles buried in a polymer matrix based on atomic force microscopy mechanical sensing. *Ultramicroscopy* **207**, 112832 (2019).

11. H. Yoo, H. Park, S. Yoo, S. On, H. Seong, S. G. Im, J. J. Kim, Highly stacked 3D organic integrated circuits with via-hole-less multilevel metal interconnects. *Nat. Commun.* **10**, 2424 (2019).

12. K. J. Harry, D. T. Hallinan, D. Y. Parkinson, A. A. MacDowell, N. P. Balsara, Detection of subsurface structures underneath dendrites formed on cycled lithium metal electrodes. *Nat. Mater.* **13**, 69–73 (2014).

13. J. F. Yin, Q. Bai, B. Zhang, Methods for detection of subsurface damage: A review. *Chin. J. Mech. Eng.* **31**, 31–41 (2018).

14. P. Vitry, E. Bourillot, C. Plassard, Y. Lacroute, E. Calkins, L. Tetard, E. Lesniewska, Mode-synthesizing atomic force microscopy for 3D reconstruction of embedded low-density dielectric nanostructures. *Nano Res.* **8**, 2199–2205 (2015).

15. M. Soliman, Y. Ding, L. Tetard, Nanoscale subsurface imaging. *J. Phys. Condens. Matter* **29**, 173001 (2017).

16. K. Moon, H. Park, J. Kim, Y. Do, S. Lee, G. Lee, H. Kang, H. Han, Subsurface nanoimaging by broadband terahertz pulse near-field microscopy. *Nano Lett.* **15**, 549–552 (2015).

17. R. Garcia, Images from below the surface. *Nat. Nanotechnol.* **5**, 101–102 (2010).

18. A. P. Schuller, M. Wojtynek, D. Mankus, M. Tatli, R. Kronenberg-Tenga, S. G. Regmi, P. V. Dip, A. K. R. Lytton-Jean, E. J. Brignole, M. Dasso, K. Weis, O. Medalia, T. U. Schwartz, The cellular environment shapes the nuclear pore complex architecture. *Nature* **598**, 667–671 (2021).

19. R. Maiti, C. Patil, M. A. S. R. Saadi, T. Xie, J. G. Azadani, B. Uluutku, R. Amin, A. F. Briggs, M. Miscuglio, D. Van Thourhout, S. D. Solares, T. Low, R. Agarwal, S. R. Bank, V. J. Sorger, Strain-engineered high-responsivity $MoTe_2$ photodetector for silicon photonic integrated circuits. *Nat. Photonics* **14**, 578–584 (2020).

20. T. Paltrinieri, L. Bondi, V. Đerek, B. Fraboni, E. D. Głowacki, T. Cramer, Understanding photocapacitive and photofaradaic processes in organic semiconductor photoelectrodes for optobioelectronics. *Adv. Funct. Mater.* **31**, 2010116 (2021).

21. D. Kokkinis, M. Schaffner, A. R. Studart, Multimaterial magnetically assisted 3D printing of composite materials. *Nat. Commun.* **6**, 8643 (2015).

22. M. Oehzelt, N. Koch, G. Heimel, Organic semiconductor density of states controls the energy level alignment at electrode interfaces. *Nat. Commun.* **5**, 4174 (2014).

23. T. Schweinböck, S. Hommel, Quantitative scanning microwave microscopy: A calibration flow. *Microelectron. Reliab.* **54**, 2070–2074 (2014).

24. M. Ruby, B. W. Heinrich, J. I. Pascual, K. J. Franke, Experimental demonstration of a two-band superconducting state for lead using scanning tunneling spectroscopy. *Phys. Rev. Lett.* **114**, 157001 (2015).

25. A. Poddubny, I. Iorsh, P. Belov, Y. Kivshar, Hyperbolic metamaterials. *Nat. Photonics* **7**, 958–967 (2013).

26. L. Tetard, A. Passian, K. T. Venmar, R. M. Lynch, B. H. Voy, G. Shekhawat, V. P. Dravid, T. Thundat, Imaging nanoparticles in cells by nanomechanical holography. *Nat. Nanotechnol.* **3**, 501–505 (2008).

27. L. Tetard, A. Passian, R. M. Lynch, B. H. Voy, G. Shekhawat, V. Dravid, T. Thundat, Elastic phase response of silica nanoparticles buried in soft matter. *Appl. Phys. Lett.* **93**, 133113 (2008).

28. K. Bērziņš, S. J. Fraser-Miller, K. C. Gordon, Pseudo-3D subsurface imaging of pharmaceutical solid dosage forms using micro-spatially offset low-frequency Raman spectroscopy. *Anal. Chem* **93**, 8986, 8993 (2021).

29. A. Besinis, T. De Peralta, C. J. Tredwin, R. D. Handy, Review of nanomaterials in dentistry: Interactions with the oral microenvironment, clinical applications, hazards, and benefits. *ACS Nano* **9**, 2255–2289 (2015).

30. Z. Zhao, Z. Zhou, J. Bao, Z. Wang, J. Hu, X. Chi, K. Ni, R. Wang, X. Chen, Z. Chen, J. Gao, Octapod iron oxide nanoparticles as high-performance T_2 contrast agents for magnetic resonance imaging. *Nat. Commun.* **4**, 2266 (2013).

31. C. Liu, X. Liu, X. Xiang, X. Pang, S. Chen, Y. Zhang, E. Ren, L. Zhang, X. Liu, P. Lv, X. Wang, W. Luo, N. Xia, X. Chen, G. Liu, A nanovaccine for antigen self-presentation and immunosuppression reversal as a personalized cancer immunotherapy strategy. *Nat. Nanotechnol.* **17**, 531–540 (2022).

32. S. H. Park, J. Hwang, G. S. Park, J. H. Ha, M. Zhang, D. Kim, D. J. Yun, S. Lee, S. H. Lee, Modeling the electrical resistivity of polymer composites with segregated structures. *Nat. Commun.* **10**, 2537 (2019).

33. I. A. Kinloch, J. Suhr, J. Lou, R. J. Young, P. M. Ajayan, Composites with carbon nanotubes and graphene: An outlook. *Science* **362**, 547–553 (2018).

34. M. Hegde, L. Yang, F. Vita, R. J. Fox, R. van de Watering, B. Norder, U. Lafont, O. Francescangeli, L. A. Madsen, S. J. Picken, E. T. Samulski, T. J. Dingemans, Strong graphene oxide nanocomposites from aqueous hybrid liquid crystals. *Nat. Commun.* **11**, 830 (2020).

35. W. Hao, M. Wang, F. Zhou, H. Luo, X. Xie, F. Luo, R. Cha, A review on nanocellulose as a lightweight filler of polyolefin composites. *Carbohydr. Polym.* **243**, 116466 (2020).

36. N. Bitinis, M. Hernandez, R. Verdejo, J. M. Kenny, M. A. Lopez-Manchado, Recent advances in clay/polymer nanocomposites. *Adv. Mater.* **23**, 5229–5236 (2011).

37. R. Mangal, S. Srivastava, L. A. Archer, Phase stability and dynamics of entangled polymer-nanoparticle composites. *Nat. Commun.* **6**, 7198 (2015).

38. R. Garcia, Nanomechanical mapping of soft materials with the atomic force microscope: Methods, theory and applications. *Chem. Soc. Rev.* **49**, 5850–5884 (2020).

39. R. García, R. Pérez, Dynamic atomic force microscopy methods. *Surf. Sci. Rep.* **47**, 197–301 (2002).

40. D. Ebeling, B. Eslami, S. D. J. Solares, Visualizing the subsurface of soft matter: Simultaneous topographical imaging, depth modulation, and compositional mapping with triple frequency atomic force microscopy. *ACS Nano* **7**, 10387–10396 (2013).

41. A. P. Perrino, Y. K. Ryu, C. A. Amo, M. P. Morales, R. Garcia, Subsurface imaging of silicon nanowire circuits and iron oxide nanoparticles with sub-10 nm spatial resolution. *Nanotechnology* **27**, 275703 (2016).

42. R. Garcia, E. T. Herruzo, The emergence of multifrequency force microscopy. *Nat. Nanotechnol.* **7**, 217–226 (2012).

43. C. A. Amo, A. P. Perrino, A. F. Payam, R. Garcia, Mapping elastic properties of heterogeneous materials in liquid with angstrom-scale resolution. *ACS Nano* **11**, 8650–8659 (2017).

44. V. G. Gisbert, C. A. Amo, M. Jaafar, A. Asenjo, R. Garcia, Quantitative mapping of magnetic properties at the nanoscale with bimodal AFM. *Nanoscale* **13**, 2026–2033 (2021).

45. Y. M. Efremov, D. M. Suter, P. S. Timashev, A. Raman, 3D nanomechanical mapping of subcellular and sub-nuclear structures of living cells by multi-harmonic AFM with long-tip microcantilevers. *Sci. Rep.* **12**, 529 (2022).

46. T. Wang, C. Ma, W. Hu, Y. Chen, J. Chu, Visualizing subsurface defects in graphite by acoustic atomic force microscopy. *Microsc. Res. Tech.* **80**, 66–74 (2016).

47. C. Tinker-Mill, J. Mayes, D. Allsop, O. V. Kolosov, Ultrasonic force microscopy for nanomechanical characterization of early and late-stage amyloid-β peptide aggregation. *Sci. Rep.* **4**, 4004 (2014).

48. F. Bin Li, G. E. Thompson, R. C. Newman, Force modulation atomic force microscopy: Background, development and application to electrodeposited cerium oxide films. *Appl. Surf. Sci.* **126**, 21–33 (1998).

49. S. M. Deese, L. E. Englade-Franklin, L. J. Hill, J. Pyun, J. Y. Chan, J. C. Garno, Subsurface imaging of the cores of polymer-encapsulated cobalt nanoparticles using force modulation microscopy. *J. Phys. Chem. C* **121**, 23498–23504 (2017).

50. U. Rabe, W. Arnold, Acoustic microscopy by atomic force microscopy. *Appl. Phys. Lett.* **64**, 1493–1495 (1994).

51. N. A. Burnham, Scanning local-acceleration microscopy. *J. Vac. Sci. Technol. B* **14**, 794–799 (1996).

52. S. Hu, C. Su, W. Arnold, Imaging of subsurface structures using atomic force acoustic microscopy at GHz frequencies. *J. Appl. Phys.* **109**, 084324 (2011).

53. J. T. Zeng, K. Y. Zhao, H. R. Zeng, H. Z. Song, L. Y. Zheng, G. R. Li, Q. R. Yin, Subsurface defect of amorphous carbon film imaged by near field acoustic microscopy. *Appl. Phys. A* **91**, 261–265 (2008).

54. X. Li, A. Lu, W. Deng, L. Su, J. Wang, M. Ding, Noninvasive subcellular imaging using atomic force acoustic microscopy (AFAM). *Cell* **8**, 314 (2019).

55. X. Yan, W. Xu, Q. Cheng, Z. Xu, Depth sensitivity of subsurface imaging using atomic force acoustic microscopy: FEA study. *J. Phys. Commun.* **2**, 115021 (2018).

56. F. Dinelli, P. Pingue, N. D. Kay, O. V. Kolosov, Subsurface imaging of two-dimensional materials at the nanoscale. *Nanotechnology* **28**, 085706 (2017).

57. B. J. Robinson, O. V. Kolosov, Probing nanoscale graphene-liquid interfacial interactions via ultrasonic force spectroscopy. *Nanoscale* **6**, 10806–10816 (2014).

58. K. Yamanaka, H. Ogiso, O. Kolosov, Ultrasonic force microscopy for nanometer resolution subsurface imaging. *Appl. Phys. Lett.* **64**, 178–180 (1994).

59. K. Yamanaka, UFM observation of lattice defects in highly oriented pyrolytic graphite. *Thin Solid Films* **273**, 116–121 (1996).

60. M. H. van Es, A. Mohtashami, R. M. T. Thijssen, D. Piras, P. L. M. J. van Neer, H. Sadeghian, Mapping buried nanostructures using subsurface ultrasonic resonance force microscopy. *Ultramicroscopy* **184**, 209–216 (2018).

61. D. Piras, P. L. M. J. van Neer, R. M. T. Thijssen, H. Sadeghian, On the resolution of subsurface atomic force microscopy and its implications for subsurface feature sizing. *Rev. Sci. Instrum.* **91**, 083702 (2020).

62. K. Kimura, K. Kobayashi, K. Matsushige, H. Yamada, Imaging of Au nanoparticles deeply buried in polymer matrix by various atomic force microscopy techniques. *Ultramicroscopy* **133**, 41–49 (2013).

63. J. P. Killgore, J. Y. Kelly, C. M. Stafford, M. J. Fasolka, D. C. Hurley, Quantitative subsurface contact resonance force microscopy of model polymer nanocomposites. *Nanotechnology* **22**, 175706 (2011).

64. M. Reggente, D. Passeri, L. Angeloni, F. A. Scaramuzzo, M. Barteri, F. De Angelis, I. Persiconi, M. E. De Stefano, M. Rossi, Detection of stiff nanoparticles within cellular structures by contact resonance atomic force microscopy subsurface nanomechanical imaging. *Nanoscale* **9**, 5671–5676 (2017).

65. Q. Tu, B. Lange, Z. Parlak, J. M. J. Lopes, V. Blum, S. Zauscher, Quantitative subsurface atomic structure fingerprint for 2D materials and heterostructures by first-principles-calibrated contact-resonance atomic force microscopy. *ACS Nano* **10**, 6491–6500 (2016).

66. G. Stan, S. D. Solares, B. Pittenger, N. Erina, C. Su, Nanoscale mechanics by tomographic contact resonance atomic force microscopy. *Nanoscale* **6**, 962–969 (2014).

67. W. Wang, C. Ma, Y. Chen, L. Zheng, H. Liu, J. Chu, Subsurface imaging of flexible circuits via contact resonance atomic force microscopy. *Beilstein J. Nanotechnol.* **10**, 1636–1647 (2019).

68. H. J. Sharahi, M. Janmaleki, L. Tetard, S. Kim, H. Sadeghian, G. J. Verbiest, Acoustic subsurface-atomic force microscopy: Three-dimensional imaging at the nanoscale. *J. Appl. Phys.* **129**, 030901 (2021).

69. G. J. Verbiest, T. H. Oosterkamp, M. J. Rost, Cantilever dynamics in heterodyne force microscopy. *Ultramicroscopy* **135**, 113–120 (2013).

70. G. J. Verbiest, T. H. Oosterkamp, M. J. Rost, Subsurface-AFM: Sensitivity to the heterodyne signal. *Nanotechnology* **24**, 365701 (2013).

71. G. J. Verbiest, M. J. Rost, Beating beats mixing in heterodyne detection schemes. *Nat. Commun.* **6**, 6444 (2015).

72. M. T. Cuberes, H. E. Assender, G. A. D. Briggs, O. V. Kolosov, Heterodyne force microscopy of PMMA/rubber nanocomposites: Nanomapping of viscoelastic response at ultrasonic frequencies. *J. Phys. D Appl. Phys.* **33**, 2347–2355 (2000).

73. G. S. Shekhawat, V. P. Dravid, Nanoscale imaging of buried structures via scanning near-field ultrasound holography. *Science* **310**, 89–92 (2005).

74. A. C. Diebold, Subsurface imaging with scanning ultrasound holography. *Science* **310**, 61–62 (2005).

75. G. Stan, E. Mays, H. J. Yoo, S. W. King, Nanoscale tomographic reconstruction of the subsurface mechanical properties of low-k high-aspect ratio patterns. *Nanotechnology* **27**, 485706 (2016).

76. G. Stan, R. S. Gates, Intermittent contact resonance atomic force microscopy. *Nanotechnology* **25**, 245702 (2014).

77. L. Tetard, A. Passian, T. Thundat, New modes for subsurface atomic force microscopy through nanomechanical coupling. *Nat. Nanotechnol.* **5**, 105–109 (2010).

78. L. Tetard, A. Passian, R. H. Farahi, T. Thundat, Atomic force microscopy of silica nanoparticles and carbon nanohorns in macrophages and red blood cells. *Ultramicroscopy* **110**, 586–591 (2010).

79. P. Vitry, E. Bourillot, C. Plassard, Y. Lacroute, L. Tetard, E. Lesniewska, Advances in quantitative nanoscale subsurface imaging by mode-synthesizing atomic force microscopy. *Appl. Phys. Lett.* **105**, 053110 (2014).

80. L. Tetard, A. Passian, R. H. Farahi, T. Thundat, B. H. Davison, Opto-nanomechanical spectroscopic material characterization. *Nat. Nanotechnol.* **10**, 870–877 (2015).

81. H. T. Thompson, F. Barroso-Bujans, J. G. Herrero, R. Reifenberger, A. Raman, Subsurface imaging of carbon nanotube networks in polymers with DC-biased multifrequency dynamic atomic force microscopy. *Nanotechnology* **24**, 135701 (2013).

82. M. Zhao, X. Gu, S. E. Lowther, C. Park, Y. C. Jean, T. Nguyen, Subsurface characterization of carbon nanotubes in polymer composites via quantitative electric force microscopy. *Nanotechnology* **21**, 225702 (2010).

83. T. S. Jespersen, J. Nygard, Mapping of individual carbon nanotubes in polymer/nanotube composites using electrostatic force microscopy. *Appl. Phys. Lett.* **90**, 183108 (2007).

84. M. J. Cadena, R. Misiego, K. C. Smith, A. Avila, B. Pipes, R. Reifenberger, A. Raman, Sub-surface imaging of carbon nanotube-polymer composites using dynamic AFM methods. *Nanotechnology* **24**, 135706 (2013).

85. J. W. Li, J. P. Cleveland, R. Proksch, Bimodal magnetic force microscopy: Separation of short and long range forces. *Appl. Phys. Lett.* **94**, 163118 (2009).

86. O. A. Castañeda-Uribe, R. Reifenberger, A. Raman, A. Avila, Depth-sensitive subsurface imaging of polymer nanocomposites using second harmonic Kelvin probe force microscopy. *ACS Nano* **9**, 2938–2947 (2015).

87. D. Passeri, C. Dong, M. Reggente, L. Angeloni, M. Barteri, F. A. Scaramuzzo, F. de Angelis, F. Marinelli, F. Antonelli, F. Rinaldi, C. Marianecci, M. Carafa, A. Sorbo, D. Sordi, I. W. C. E. Arends, M. Rossi, Magnetic force microscopy: Quantitative issues in biomaterials. *Biomatter* **4**, e29507 (2014).

88. L. Angeloni, M. Reggente, D. Passeri, M. Natali, M. Rossi, Identification of nanoparticles and nanosystems in biological matrices with scanning probe microscopy. *Wiley Interdiscip. Rev. Nanomed. Nanobiotechnol.* **10**, e1521 (2018).

89. T. M. Nocera, Y. Zeng, G. Agarwal, Distinguishing ferritin from apoferritin using magnetic force microscopy. *Nanotechnology* **25**, 461001 (2014).

90. A. R. Blissett, B. Ollander, B. Penn, D. M. McTigue, G. Agarwal, Magnetic mapping of iron in rodent spleen. *Nanomed. Nanotechnol. Biol. Med.* **13**, 977–986 (2017).

91. Z. Wang, A. Cuschieri, Tumour cell labelling by magnetic nanoparticles with determination of intracellular iron content and spatial distribution of the intracellular iron. *Int. J. Mol. Sci.* **14**, 9111–9125 (2013).

92. C. Dong, S. Corsetti, D. Passeri, M. Rossi, M. Carafa, F. Pantanella, F. Rinaldi, C. Ingallina, A. Sorbo, C. Marianecci, Visualization and quantification of magnetic nanoparticles into vesicular systems by combined atomic and magnetic force microscopy, in *AIP Conference Proceedings*, 23 June 2015 (AIP Publishing LLC, 2015), vol. 1667, p. 020011.

93. G. Gramse, A. Kölker, T. Lim, T. J. Z. Stock, H. Solanki, S. R. Schofield, E. Brinciotti, G. Aeppli, F. Kienberger, N. J. Curson, Nondestructive imaging of atomically thin nanostructures buried in silicon. *Sci. Adv.* **3**, e1602586 (2017).

94. G. Gramse, E. Brinciotti, A. Lucibello, S. B. Patil, M. Kasper, C. Rankl, R. Giridharagopal, P. Hinterdorfer, R. Marcelli, F. Kienberger, Quantitative sub-surface and non-contact imaging using scanning microwave microscopy. *Nanotechnology* **26**, 135701 (2015).

95. G. Gramse, M. Kasper, L. Fumagalli, G. Gomila, P. Hinterdorfer, F. Kienberger, Calibrated complex impedance and permittivity measurements with scanning microwave microscopy. *Nanotechnology* **25**, 145703 (2014).

96. C. Plassard, E. Bourillot, J. Rossignol, Y. Lacroute, E. Lepleux, L. Pacheco, E. Lesniewska, Detection of defects buried in metallic samples by scanning microwave microscopy. *Phys. Rev. B.* **83**, 121409–5 (2011).

97. K. Lai, W. Kundhikanjana, H. Peng, Y. Cui, M. A. Kelly, Z. X. Shen, Tapping mode microwave impedance microscopy. *Rev. Sci. Instrum.* **80**, 043707 (2009).

98. M. C. Biagi, R. Fabregas, G. Gramse, M. Van Der Hofstadt, A. Juárez, F. Kienberger, L. Fumagalli, G. Gomila, Nanoscale electric permittivity of single bacterial cells at gigahertz frequencies by scanning microwave microscopy. *ACS Nano* **10**, 280–288 (2016).

99. A. Tselev, J. Velmurugan, A. V. Ievlev, S. V. Kalinin, A. Kolmakov, Seeing through walls at the nanoscale: Microwave microscopy of enclosed objects and processes in liquids. *ACS Nano* **10**, 3562–3570 (2016).

100. L. You, J. J. Ahn, Y. S. Obeng, J. J. Kopanski, Subsurface imaging of metal lines embedded in a dielectric with a scanning microwave microscope. *J. Phys. D Appl. Phys.* **49**, 45502 (2015).

101. E. Brinciotti, G. Gramse, S. Hommel, T. Schweinboeck, A. Altes, M. A. Fenner, J. Smoliner, M. Kasper, G. Badino, S. S. Tuca, F. Kienberger, Probing resistivity and doping concentration of semiconductors at the nanoscale using scanning microwave microscopy. *Nanoscale* **7**, 14715–14722 (2015).

102. A. O. Oladipo, A. Lucibello, M. Kasper, S. Lavdas, G. M. Sardi, E. Proietti, F. Kienberger, R. Marcelli, N. C. Panoiu, Analysis of a transmission mode scanning microwave microscope for subsurface imaging at the nanoscale. *Appl. Phys. Lett.* **105**, 133112 (2014).

103. H. P. Huber, I. Humer, M. Hochleitner, M. Fenner, M. Moertelmaier, C. Rankl, A. Imtiaz, T. M. Wallis, H. Tanbakuchi, P. Hinterdorfer, P. Kabos, J. Smoliner, J. J. Kopanski, F. Kienberger, Calibrated nanoscale dopant profiling using a scanning microwave microscope. *J. Appl. Phys.* **111**, 014301 (2012).

104. L. Bozec, M. Odlyha, Thermal denaturation studies of collagen by microthermal analysis and atomic force microscopy. *Biophys. J.* **101**, 228–236 (2011).

105. G. Mills, J. M. R. Weaver, G. Harris, W. Chen, J. Carrejo, L. Johnson, B. Rogers, Detection of subsurface voids using scanning thermal microscopy. *Ultramicroscopy* **80**, 7–11 (1999).

106. Y. Zhang, W. Zhu, F. Hui, M. Lanza, T. Borca-Tasciuc, M. Muñoz Rojo, A review on principles and applications of scanning thermal microscopy (SThM). *Adv. Funct. Mater.* **30**, 1900892 (2020).

107. Y. Zhang, E. E. Castillo, R. J. Mehta, G. Ramanath, T. Borca-Tasciuc, A noncontact thermal microprobe for local thermal conductivity measurement. *Rev. Sci. Instrum.* **82**, 024902 (2011).

108. S. Cho, S. D. Kang, W. Kim, E. S. Lee, S. J. Woo, K. J. Kong, I. Kim, H. Do Kim, T. Zhang, J. A. Stroscio, Y. H. Kim, H. K. Lyeo, Thermoelectric imaging of structural disorder in epitaxial graphene. *Nat. Mater.* **12**, 913–918 (2013).

109. E. Loftus, M. C. Anderson, C. Green, J. F. Kihlstrom, B. E. Depue, M. T. Banich, T. Curran, B. A. Van Der Kolk, J. A. Burbridge, J. Suzuki, C. Purdon, E. T. Rolls, W. L. Thompson, M. S. Gazzaniga, R. E. Passingham, A. M. Owen, G. Calcaterra, P. Bourgouin, C. M. Kelley, B. D. Mcelree, S. Chaiken, Y. Trope, T. J. Ross, E. A. Stein, T. W. Robbins, R. A. Poldrack, M. D. Lieberman, K. D. Williams, K. Meyer, V. Treyer, E. Fehr, D. Singel, Scattering and interference in epitaxial graphene. *Science* **317**, 219–223 (2007).

110. F. Menges, P. Mensch, H. Schmid, H. Riel, A. Stemmer, B. Gotsmann, Temperature mapping of operating nanoscale devices by scanning probe thermometry. *Nat. Commun.* **7**, 1–6 (2016).

111. F. Menges, H. Riel, A. Stemmer, C. Dimitrakopoulos, B. Gotsmann, Thermal transport into graphene through nanoscopic contacts. *Phys. Rev. Lett.* **111**, 205901 (2013).

112. K. Q. Xu, H. R. Zeng, K. Y. Zhao, G. R. Li, X. Shi, L. D. Chen, Scanning near-field thermoelectric microscopy for subsurface nanoscale thermoelectric behavior. *Appl. Phys. A* **122**, 521 (2016).

113. A. F. Payam, W. Trewby, K. Voïtchovsky, Simultaneous viscosity and density measurement of small volumes of liquids using a vibrating microcantilever. *Analyst* **142**, 1492–1498 (2017).

114. A. Yao, K. Kobayashi, S. Nosaka, K. Kimura, H. Yamada, Visualization of Au nanoparticles buried in a polymer matrix by scanning thermal noise microscopy. *Sci. Rep.* **7**, 42718 (2017).

115. A. F. Payam, D. Martin-Jimenez, R. Garcia, Force reconstruction from tapping mode force microscopy experiments. *Nanotechnology* **26**, 185706 (2015).

116. N. Bhalla, Y. Pan, Z. Yang, A. F. Payam, Opportunities and challenges for biosensors and nanoscale analytical tools for pandemics: COVID-19. *ACS Nano* **14**, 7783–7807 (2020).

117. C. Roduit, S. Sekatski, G. Dietler, S. Catsicas, F. Lafont, S. Kasas, Stiffness tomography by atomic force microscopy. *Biophys. J.* **97**, 674–677 (2009).

118. L. Stühn, A. Fritschen, J. Choy, M. Dehnert, C. Dietz, Nanomechanical sub-surface mapping of living biological cells by force microscopy. *Nanoscale* **11**, 13089–13097 (2019).

119. C. Roduit, G. Longo, I. Benmessaoud, A. Volterra, B. Saha, G. Dietler, S. Kasas, Stiffness tomography exploration of living and fixed macrophages. *J. Mol. Recognit.* **25**, 241–246 (2012).

120. G. Longo, L. M. Rio, C. Roduit, A. Trampuz, A. Bizzini, G. Dietler, S. Kasas, Force volume and stiffness tomography investigation on the dynamics of stiff material under bacterial membranes. *J. Mol. Recognit.* **25**, 278–284 (2012).

121. K. Radotić, C. Roduit, J. Simonović, P. Hornitschek, C. Fankhauser, D. Mutavdžić, G. Steinbach, G. Dietler, S. Kasas, Atomic force microscopy stiffness tomography on living arabidopsis thaliana cells

reveals the mechanical properties of surface and deep cell-wall layers during growth. *Biophys. J.* **103**, 386–394 (2012).

122. M. Penedo, K. Miyazawa, N. Okano, H. Furusho, T. Ichikawa, M. S. Alam, K. Miyata, C. Nakamura, T. Fukuma, Visualizing intracellular nanostructures of living cells by nanoendoscopy-AFM. *Sci. Adv.* **7**, eabj4990 (2021).

123. A. Krull, P. Hirsch, C. Rother, A. Schiffrin, C. Krull, Artificial-intelligence-driven scanning probe microscopy. *Commun. Phys.* **3**, 54 (2020).

124. M. Rashidi, R. A. Wolkow, Autonomous scanning probe microscopy in situ tip conditioning through machine learning. *ACS Nano* **12**, 5185–5189 (2018).

125. R. Garcia, Intracellular forces from stiffness. *Nat. Mater.* **18**, 1037–1038 (2019).

126. T. Ando, High-speed atomic force microscopy and its future prospects. *Biophys. Rev.* **10**, 285–292 (2018).

127. A. Belianinov, S. V. Kalinin, S. Jesse, Complete information acquisition in dynamic force microscopy. *Nat. Commun.* **6**, 6550 (2015).

128. A. F. Payam, P. Biglarbeigi, A. Morelli, P. Lemoine, J. McLaughlin, D. Finlay, Data acquisition and imaging using wavelet transform: A new path for high speed transient force microscopy. *Nanoscale Adv.* **3**, 383–398 (2021).

129. G. Vasilakis, H. Shen, K. Jensen, M. Balabas, D. Salart, B. Chen, E. S. Polzik, Generation of a squeezed state of an oscillator by stroboscopic back-action-evading measurement. *Nat. Phys.* **11**, 389–392 (2015).

130. A. Passian, G. Siopsis, Quantum state atomic force microscopy. *Phys. Rev. A* **95**, 043812 (2017).

131. L. Tetard, A. Passian, R. H. Farahi, B. H. Davison, S. Jung, A. J. Ragauskas, A. L. Lereu, T. Thundat, Nanometrology of delignified Populus using mode synthesizing atomic force microscopy. *Nanotechnology* **22**, 465702 (2011).

132. G. Shekhawat, A. Srivastava, S. Avasthy, V. Dravid, Ultrasound holography for noninvasive imaging of buried defects and interfaces for advanced interconnect architectures. *Appl. Phys. Lett.* **95**, 2007–2010 (2009).

133. Y. Zhang, M. Yang, M. Ozkan, C. S. Ozkan, Magnetic force microscopy of iron oxide nanoparticles and their cellular uptake. *Biotechnol. Prog.* **25**, 923–928 (2009).

134. H.-b. Shen, D.-h. Long, L.-z. Zhu, X.-y. Li, Y.-m. Dong, N.-q. Jia, H.-q. Zhou, X. Xin, Y. Sun, Magnetic force microscopy analysis of apoptosis of HL-60 cells induced by complex of antisense oligonucleotides and magnetic nanoparticles. *Biophys. Chem.* **122**, 1–4 (2006).

135. Y. J. Oh, H. P. Huber, M. Hochleitner, M. Duman, B. Bozna, M. Kastner, F. Kienberger, P. Hinterdorfer, High-frequency electromagnetic dynamics properties of THP1 cells using scanning microwave microscopy. *Ultramicroscopy* **111**, 1625–1629 (2011).

136. S. S. Tuca, G. Badino, G. Gramse, E. Brinciotti, M. Kasper, Y. J. Oh, R. Zhu, C. Rankl, P. Hinterdorfer, F. Kienberger, Calibrated complex impedance of CHO cells and E. coli bacteria at GHz frequencies using scanning microwave microscopy. *Nanotechnology* **27**, 135702 (2016).

137. H. Takano, S. S. Wong, J. A. Harnisch, M. D. Porter, Mapping the subsurface composition of organic films by electric force microscopy. *Langmuir* **16**, 5231–5233 (2000).

138. N. D. Kay, B. J. Robinson, V. I. Fal'Ko, K. S. Novoselov, O. V. Kolosov, Electromechanical sensing of substrate charge hidden under atomic 2D crystals. *Nano Lett.* **14**, 3400–3404 (2014).

139. M. M. Marzec, K. Awsiuk, A. Bernasik, J. Rysz, J. Haberko, W. Łuzny, A. Budkowski, Buried polymer/metal interfaces examined with Kelvin Probe Force Microscopy. *Thin Solid Films* **531**, 271–276 (2013).

140. A. P. McGuigan, B. D. Huey, G. A. D. Briggs, O. V. Kolosov, Y. Tsukahara, M. Yanaka, Measurement of debonding in cracked nanocomposite films by ultrasonic force microscopy. *Appl. Phys. Lett.* **80**, 1180–1182 (2002).

141. H. J. Sharahi, G. Shekhawat, V. Dravid, S. Park, P. Egberts, S. Kim, Contrast mechanisms on nanoscale subsurface imaging in ultrasonic AFM: Scattering of ultrasonic waves and contact stiffness of the tip-sample. *Nanoscale* **9**, 2330–2339 (2017).

142. A. Buck, B. K. Jones, H. M. Pollock, Temperature and thermal conductivity modes of scanning probe microscopy for electromigration studies. *Microelectron. Reliab.* **37**, 1495–1498 (1997).
